# Discovery of Unique Lanthionine Synthetases Reveals New Mechanistic and Evolutionary Insights

**DOI:** 10.1371/journal.pbio.1000339

**Published:** 2010-03-23

**Authors:** Yuki Goto, Bo Li, Jan Claesen, Yanxiang Shi, Mervyn J. Bibb, Wilfred A. van der Donk

**Affiliations:** 1 Department of Chemistry, University of Illinois at Urbana-Champaign, Urbana, Illinois, United States of America; 2 Department of Biochemistry, University of Illinois at Urbana-Champaign, Urbana, Illinois, United States of America; 3 Department of Molecular Microbiology, John Innes Centre, Norwich, United Kingdom; 4 The Howard Hughes Medical Institute, University of Illinois at Urbana-Champaign, Urbana, Illinois, United States of America; Stanford University, United States of America

## Abstract

Lantibiotic synthetases are remarkable biocatalysts generating conformationally constrained peptides with a variety of biological activities by repeatedly utilizing two simple posttranslational modification reactions: dehydration of Ser/Thr residues and intramolecular addition of Cys thiols to the resulting dehydro amino acids. Since previously reported lantibiotic synthetases show no apparent homology with any other known protein families, the molecular mechanisms and evolutionary origin of these enzymes are unknown. In this study, we present a novel class of lanthionine synthetases, termed LanL, that consist of three distinct catalytic domains and demonstrate in vitro enzyme activity of a family member from *Streptomyces venezuelae*. Analysis of individually expressed and purified domains shows that LanL enzymes install dehydroamino acids via phosphorylation of Ser/Thr residues by a protein kinase domain and subsequent elimination of the phosphate by a phosphoSer/Thr lyase domain. The latter has sequence homology with the phosphothreonine lyases found in various pathogenic bacteria that inactivate host mitogen activated protein kinases. A LanC-like cyclase domain then catalyzes the addition of Cys residues to the dehydro amino acids to form the characteristic thioether rings. We propose that LanL enzymes have evolved from stand-alone protein Ser/Thr kinases, phosphoSer/Thr lyases, and enzymes catalyzing thiol alkylation. We also demonstrate that the genes for all three pathways to lanthionine-containing peptides are widespread in Nature. Given the remarkable efficiency of formation of lanthionine-containing polycyclic peptides and the latter's high degree of specificity for their cognate cellular targets, it is perhaps not surprising that (at least) three distinct families of polypeptide sequences have evolved to access this structurally and functionally diverse class of compounds.

## Abstract 2

Identification of a new class of lanthionine synthetases provides insight into the mechanism and evolution of cyclic peptide biosynthesis.

## Author Summary

Many bacteria generate cyclic peptides, which have improved biological activities compared to linear peptides, including higher stability. Lanthionine-containing peptides are one such group, and different members of this group have antibiotic, anti-inflammatory, and anti-viral activities. For example, one lanthionine-containing peptide called nisin has been used to protect food items from harmful bacteria. Two different pathways for the biosynthesis of lanthionine-containing peptides have been described previously. By comparing the DNA sequences of bacterial genomes we reveal a third biosynthetic route that provides further insight into how the biosynthetic pathways for these cyclic peptides have evolved. We characterized the novel lanthionine synthetase utilized in this third pathway in the soil bacterium *Streptomyces venezuelae* and show that the purified enzyme catalyzes the chemical reactions necessary to turn a linear peptide into a peptide with multiple rings. The discovery of this third biosynthetic pathway widens the scope for the engineering of new lanthionine-containing peptides for potential use in human therapeutics.

## Introduction

Macrocyclization is a common strategy to constrain the conformational flexibility of natural peptides of both ribosomal and nonribosomal origin [1], thereby conferring increased proteolytic stability and improved affinity for their targets. Lantibiotic synthetases are remarkable catalysts that achieve macrocyclization by utilizing two simple posttranslational modification reactions, dehydration of Ser/Thr residues and subsequent intramolecular addition of Cys thiols to the dehydro amino acids to generate thioether crosslinks called (methyl)lanthionines [2]. The resulting polycyclic products have high affinity for their various targets, which to date all consist of small molecules [3]. For instance, nisin binds with high affinity to the bacterial cell wall precursor lipid II [4], and cinnamycin specifically recognizes phosphatidyl ethanolamine [5]. Nisin is the most studied lantibiotic [6] and has been used commercially to combat food-borne pathogens for 40 years in more than 80 countries without widespread development of resistance. A remarkable feature of lantibiotic biosynthesis is the extraordinary efficiency by which one or two enzymes typically generate 3–5 rings from a linear precursor peptide. How these exceptional catalysts carry out their reactions with apparently high promiscuity and yet a high degree of control and what their evolutionary origin is has not been clear since they have no obvious homology with other protein families in the databases. In this study, we report the discovery of a new class of lanthionine synthetases that provides important new insights into both their mechanisms of catalysis as well as their likely evolutionary origin.

**Figure 1:**
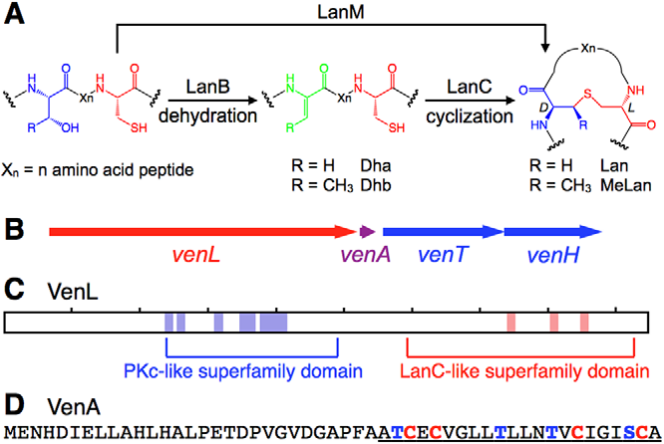
Putative lantibiotic biosynthesis in *S. venezuelae*. (A) Posttranslational modification of precursor peptides by lantibiotic synthetases. Following ribosomal synthesis of the precursor peptides, LanB or LanM enzymes dehydrate Ser and Thr to afford Dha and Dhb, respectively. Subsequently, LanC or LanM catalyze intramolecular addition of Cys thiols onto the dehydro amino acids in a stereo- and regio-selective manner to form Lan and MeLan. (B) The biosynthetic gene cluster of *S. venezuelae* consists of the synthetase gene *venL*, the precursor gene *venA*, and two components of an ABC transporter *venT* and *venH*, encoding the ATP-binding and permease subunits, respectively. (C) A conserved domain search for VenL identified a putative protein kinase domain and LanC-like domain shown in blue and red, respectively. Location of conserved residues in the domains is shown as colored boxes. (D) Primary sequence of VenA. The cysteine residues and possible dehydration sites are highlighted in red and blue, respectively. The putative core region is underlined.

Lantibiotics have been categorized into two classes based on their biosynthetic pathways [7]. For class I lantibiotics, LanB dehydratases convert Ser and Thr present in precursor peptides to dehydroalanine (Dha) and *Z*-dehydrobutyrine (Dhb), respectively. Subsequent intramolecular Michael addition of Cys thiols to Dha/Dhb catalyzed by LanC cyclases form the characteristic lanthionine (Lan, from Ser) and methyllanthionine (MeLan, from Thr) thioether crosslinks (Figure 1A). Class II lantibiotics are produced by bi-functional LanM modifying enzymes, which are responsible for both dehydration and cyclization [8]. The C-terminal cyclase domain of LanM proteins has sequence homology with the LanC enzymes, but the N-terminal dehydratase domain of LanM proteins has no homology with LanB enzymes [9]. Recent X-ray structure analysis and mutagenesis studies of LanC enzymes and LanC-like domains in LanM proteins have provided structural and mechanistic insights into the cyclization steps in lantibiotic biosynthesis [10]–[13]. In contrast, the molecular mechanism of the dehydration reaction by lantibiotic synthetases remains an open question. In this paper, we conducted a search of genome databases for putative alternative lantibiotic synthetases and report the discovery of a new biosynthetic route to lantibiotic-like peptides. We demonstrate in vitro activity for one member from a cryptic gene cluster in the differentiating mycelial soil bacterium *Streptomyces venezuelae*. This new class of enzymes provides a rare glimpse into an evolutionary path leading to lanthionine-containing peptides. Moreover, we show that the genes for all three pathways to lanthionine-containing peptides are widespread in nature and that they are not restricted to Gram-positive bacteria as long believed.

## Results

### A Putative Lantibiotic Gene Cluster in *Streptomyces venezuelae*

Analysis of the draft genome sequence of *S. venezuelae* revealed a lantibiotic-like gene cluster (Figure 1B) with a gene encoding a putative and unusual bifunctional lantibiotic synthetase with an N-terminal region (residues 225–480) resembling a serine/threonine kinase instead of the dehydratase domain found in LanM enzymes. At its C-terminus, the protein contains a LanC-like cyclization domain (residues 540–930) (Figure 1C). A nearby small open reading frame has all the hallmarks of a putative lantibiotic precursor gene including a series of Cys, Ser, and Thr residues that are localized in the C-terminal part of the gene product (Figure 1D).

A search of the publicly available databases uncovered at least nine other gene clusters that encode proteins with an N-terminal serine/threonine kinase-like domain and a C-terminal LanC-like domain (Table S1). We hypothesized that these proteins would be novel bifunctional lanthionine synthetases in which the N-terminal region containing the kinase-like domain and the C-terminal LanC-like domain would be responsible for dehydration and cyclization, respectively. Continuing the common nomenclature for lantibiotic biosynthetic genes, we refer to them as LanL proteins with the enzyme from *S. venezuelae* given the annotation VenL and its putative substrate VenA. Located immediately downstream of *venL* and *venA* (Figure 1B) are two genes, *venT* and *venH*, that appear to encode the ATP-binding and membrane permease subunits, respectively, of an ABC transporter that may be involved in export of the modified peptide out of the cell as occurs for many lantibiotics.

Interestingly, the *ven* cluster does not contain apparent immunity genes unless that role is fulfilled entirely by the putative transport genes *venTH*, which is not common for lantibiotics. Consistent with other sequenced lantibiotic gene clusters of actinomycete origin [14],[15], no genes are present encoding a protease or protease domain that might be involved in cleavage of the leader peptide. Nor does the cluster contain any regulatory genes, although the latter is not unprecedented in actinomycete antibiotic gene clusters (e.g., the erythromycin biosynthetic gene cluster of *Saccharopolyspora erythraea*) [16].

### VenL Dehydrates and Cyclizes VenA

**Figure 2:**
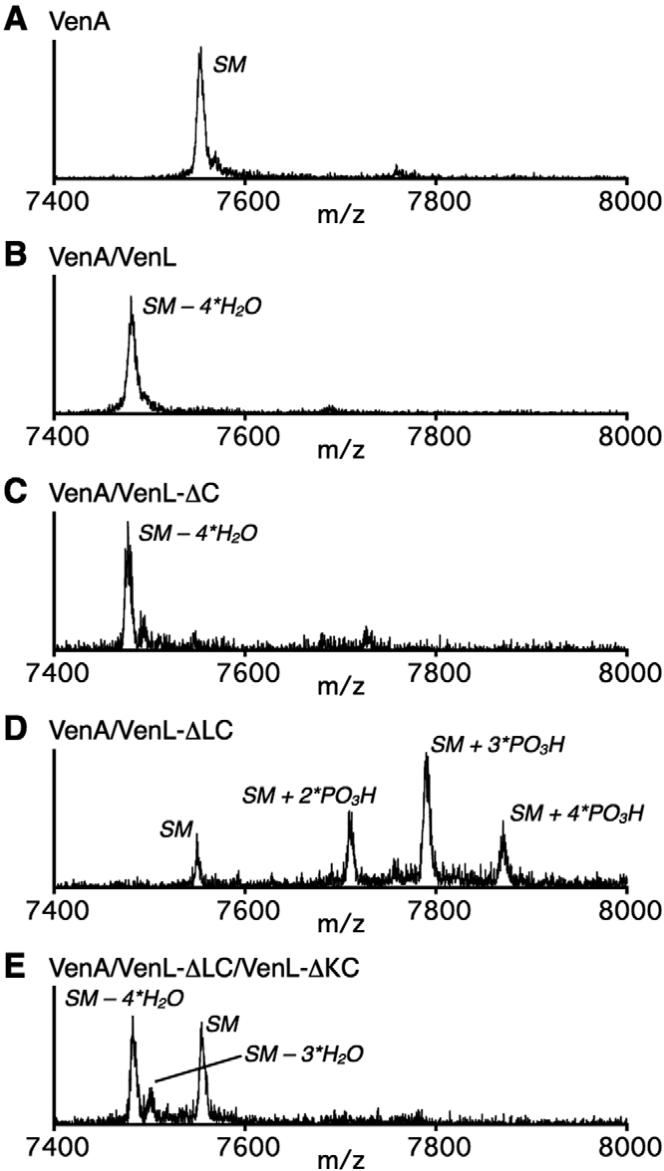
MALDI-ToF MS analysis of in vitro enzyme assays of His_6_-VenA with VenL and VenL deletion proteins. Mass spectra are depicted of (A) His_6_-VenA, (B) His_6_-VenA after incubation with His_6_-VenL, (C) His_6_-VenA after incubation with His_6_-VenL-ΔC, (D) His_6_-VenA after incubation with His_6_-VenL-ΔLC, and (E) His_6_-VenA after incubation with both His_6_-VenL-ΔLC and His_6_-VenL-ΔKC. The assignments of observed peaks are shown in the spectra, in which SM indicates the starting material (His_6_-VenA).

To verify the hypothesized function, *venL* and *venA* were cloned and heterologously expressed in *Escherichia coli*. VenA was produced as a fusion protein with an N-terminally located maltose-binding protein (MBP) and hexahistidine tag (His_6_) to improve its solubility and provide ease of purification, respectively. The fusion protein was purified by immobilized metal affinity chromatography (IMAC) and subsequently treated with tobacco etch virus protease to obtain His_6_-VenA with a predicted mass of 7,552.5 (Figure 2A). VenL was also produced with an N-terminal His_6_-tag (His_6_-VenL) and purified by IMAC and gel filtration chromatography (Figure S1). His_6_-VenA was incubated with His_6_-VenL in the presence of adenosine triphosphate (ATP), MgCl_2_, and tris(2-carboxyethyl)phosphine (TCEP) and after 3 h subjected to matrix-assisted laser desorption/ionization time-of-flight mass spectrometry (MALDI-ToF MS). After incubation with His_6_-VenL, His_6_-VenA was converted into a product with a mass corresponding to the loss of four molecules of water (−72 Da) (Figure 2B). This result clearly demonstrated that His_6_-VenL carried out the dehydration of all four Ser/Thr residues present in the putative core peptide of VenA, the region of the precursor peptide that undergoes the posttranslational modifications (underlined sequence, Figure 1C) [17]. The molecular weight of a cyclized or uncyclized product is identical, and therefore we determined the presence of any free Cys thiols in VenA after VenL treatment by reaction with iodoacetamide (IAA), a well-known thiol-selective alkylation agent. As expected, when intact His_6_-VenA was treated with IAA, its mass increased by 228 Da, corresponding to alkylation of the four Cys in the core peptide (Figure S2B). In contrast, IAA treatment of His_6_-VenA after incubation with His_6_-VenL did not result in detectable alkylation products, indicating that the great majority of free thiols were converted to thioether rings (Figure S2C). Taken together, these results demonstrate that VenL functions as a bifunctional enzyme that catalyzes both dehydration and cyclization reactions.

### The N-Terminal Region of VenL Is Responsible for Dehydration of VenA

To address the hypothesis that the N-terminal region of VenL containing the kinase-like domain would be involved in dehydration of the Ser/Thr residues in VenA, a gene encoding a truncated protein lacking the C-terminal cyclase domain of VenL (VenL-ΔC) was constructed. VenL-ΔC was expressed and purified with an N-terminal His_6_-tag and shown to catalyze the 4-fold dehydration of His_6_-VenA (Figure 2C). IAA treatment resulted in four alkylations (Figure S2D) and no detectable cyclized product lacking any of the alkylations, showing that the N-terminal region of VenL is responsible for the dehydration activity of VenL but has no or greatly reduced cyclase activity. Moreover, these findings strongly suggest that non-enzymatic cyclization is not responsible for the lack of free thiols after incubation of His_6_-VenA with full length VenL. VenL-ΔC is the first example of an in vitro reconstituted active, monofunctional peptide dehydratase.

### Sequence Alignment of LanL Enzymes with Protein Kinases and OspF Family Members

**Figure 3:**
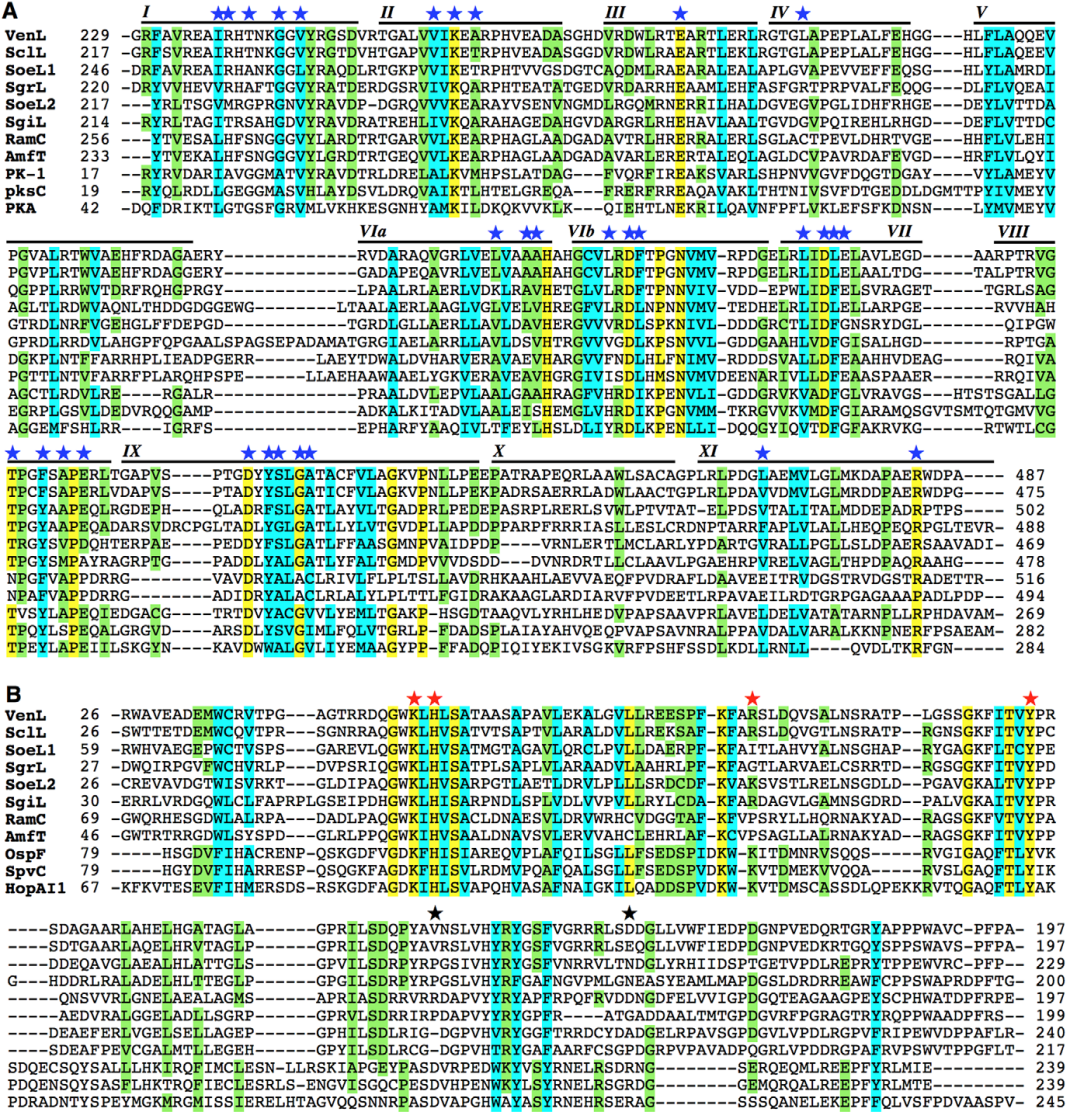
Sequence alignment of LanL protein family members with other proteins. (A) Alignment of the N-terminal regions of the LanL family with RamC, AmfT and three protein kinases (PK-1 from *S. clavuligerus* ATCC 27064, YP_002190269; PksC from *Streptomyces coelicolor* A3(2), NP_628010; and PKA from mouse, NP_032880). Fully and partially conserved residues are highlighted in yellow and green, respectively, with cyan indicating similar amino acids. Roman numerals refer to subdomains conserved across the Ser/Thr protein kinase family. Blue stars indicate the amino acids that are highly conserved in protein kinases. (B) The N-terminal regions of the LanL family, which exhibit no homology with kinases, were aligned with RamC family (RamC and AmfT) and OspF family (OspF, SpvC, and HopAI1) members. Color coding as in (A). Red stars indicate catalytic residues in the proposed mechanism of SpvC that are conserved in the LanL family, and black stars indicate proposed catalytic residues that are not found in LanL proteins. For full sequences and accession codes, see Figures S6 and S8.

To provide insights into the mechanism whereby VenL dehydrates rather than phosphorylates Ser and Thr residues, the N-terminal region of LanL proteins was aligned with RamC protein family members and known protein kinases. RamC is involved in the biosynthesis of SapB [18], a lantibiotic-like morphogenetic peptide that plays a role in sporulation in streptomycetes. RamC possesses an N-terminal kinase-like domain similar to LanL [19] but lacks a prototypical C-terminal cyclase-like domain with its characteristic zinc binding site and active site residues [2],[11],[12]. Figure 3A shows the result of sequence alignment analysis of the N-terminal regions of five LanL proteins with two RamC family proteins and three *bona fide* protein kinases. The alignment clearly indicates that the C-terminal part of the analyzed region of LanL (residues 230–487 in VenL) contains conserved kinase-like sequence motifs (blue stars, Figure 3A). On the other hand, its N-terminal part (residues 1–229 in VenL) showed no sequence homology to kinases, whereas it shares some homology with members of the RamC protein family. Based on this result, we postulated that the N-terminal part of LanL might be important for the β-elimination reaction of the phosphate group of phosphoserine/threonine to afford Dha and Dhb residues.

To test this model, additional sequence alignments were carried out of the N-terminal part of LanL and RamC with the OspF protein family (Figure 3B). Members of this family are phosphothreonine lyase effector proteins that catalyze the irreversible β-elimination reaction of a phosphate group from a phosphothreonine to produce a Dhb residue in their substrate proteins, mitogen-activated protein kinases (MAPKs) [20]–[23]. Several pathogenic bacteria use this strategy to modulate host signaling pathways. The alignment revealed that the N-terminal region of VenL possesses conserved motifs characteristic of the OspF family. In particular, four of six residues proposed as catalytic site residues in phosphothreonine lyases based on a crystal structure [21],[22] are conserved in LanL enzymes (red stars, Figure 3B). Taken together, the sequence alignment analysis suggests that LanL enzymes consist of three catalytic domains: a phosphoserine/threonine lyase domain, a Ser/Thr protein kinase domain, and a cyclase domain that act together to form Lan and MeLan.

### Catalytic Activity of VenL-ΔLC and VenL-ΔKC

To verify the hypothesis that LanL enzymes consist of three catalytic modules, we constructed two His_6_-tagged VenL truncation mutants consisting of only the kinase domain (residues 201–513, VenL-ΔLC) or only the putative phosphoserine/threonine lyase domain (residues 1–212, VenL-ΔKC). Incubation of His_6_-VenA with His_6_-VenL-ΔLC gave a series of new peaks in the mass spectrum corresponding to two, three, and four phosphorylations of VenA (Figure 2D), demonstrating the kinase activity of VenL-ΔLC. When His_6_-VenA was incubated in the presence of both His_6_-VenL-ΔLC and His_6_-VenL-ΔKC, dehydration of VenA was observed albeit with incomplete conversion (Figure 2E), showing that VenL-ΔKC can catalyze the β-elimination of phosphate groups present in phosphorylated VenA to afford Dha/Dhb residues. Collectively, these results are consistent with dehydratase activity of VenL being divided into two distinct catalytic modules, a central kinase domain and an N-terminal lyase domain.

### Investigation of the *ven* Gene Cluster

To determine the possible physiological function of VenA, both *venA* and *venL* were deleted individually from the *S. venezuelae* genome. Neither deletion had an effect on cell growth or morphological differentiation, and no difference was observed between the mutants and the wild type strain when screened against *Micrococcus luteus* ATCC4698 for antibiotic activity on 10 different solid growth media. An attempt to enhance expression of the *ven* gene cluster by introducing the strong constitutive ermE* promoter upstream of *venL* in *S. venezuelae* did not yield any phenotypic differences using the same screening conditions. MALDI-ToF analyses of culture supernatants of the wild type and mutant strains grown in different liquid media gave identical spectra, and the wild type culture failed to reveal a peptide corresponding in mass to the in vitro produced compound or to any analogs differing in the site of leader peptide cleavage, number of dehydrations, or that had retained the leader peptide. Apparently the *ven* gene cluster is not expressed in its natural host under the growth conditions used.

Consequently, cosmids containing the wild type *ven* gene cluster, as well as the *venL-* and *venA*-deleted versions, were introduced into *Streptomyces lividans* by conjugation in an attempt to obtain heterologous expression. No phenotypic differences were observed between the three ex-conjugants, and MALDI-ToF analyses of culture supernatants again failed to identify a peptide with any predicted masses. It is conceivable that some unknown environmental signal is required to activate *ven* gene expression. The absence of genes with putative regulatory and proteolytic functions and potentially self-resistance mechanisms, and the possibility that they lie elsewhere in the genome of *S. venezuelae*, might also explain the failure to detect heterologous expression of the *ven* gene cluster in *S. lividans*.

### Determination of the Ring Topology of VenL-Processed VenA

With few exceptions [24],[25], all lantibiotics known to date were first isolated and purified from natural sources. On the other hand, these investigations into VenA originated from a bioinformatic approach, and to date we have not succeeded in detecting production of the peptide by *S. venezuelae* or after cloning the gene cluster in *S. lividans*, which is often used to express heterologous gene clusters of actinomycete origin. Therefore, the structure of the mature peptide, which we have termed venezuelin, is unknown. To gain insight into the topology of the lanthionine rings in VenA after processing by VenL, a series of VenA analogs were made in which each cysteine residue was replaced with an alanine residue (VenA-C32A, VenA-C34A, VenA-C45A, and VenA-C50A). Ala29 in the leader peptide region was also replaced with a Lys in these VenA analogs to improve their solubility. Each VenA analog was incubated with His_6_-VenL followed by endoproteinase Glu-C treatment to yield the C-terminal cyclized peptide spanning Thr18-Ala51, and subsequently subjected to electron spray ionization-quadrupole/ToF (ESI-Q/ToF) MS (Figure S3).

**Figure 4:**
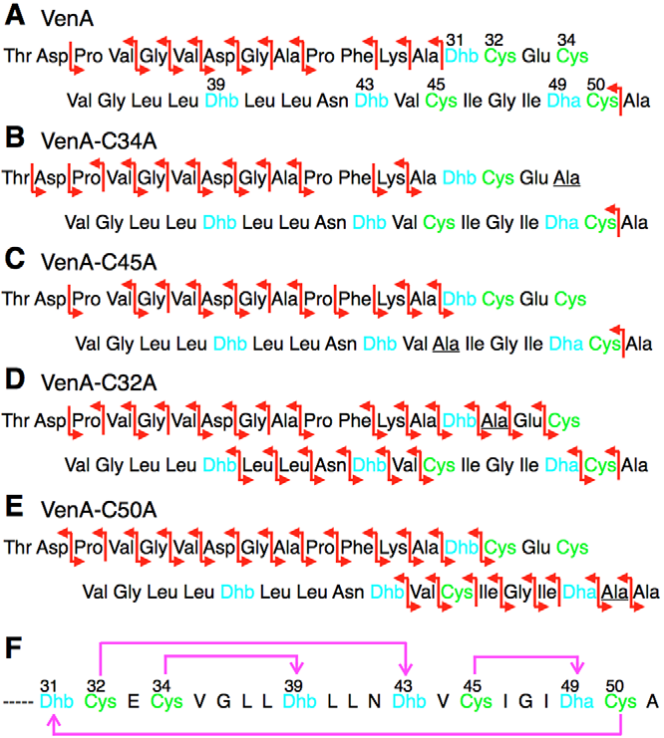
Determination of the structure of venezuelin. Red arrows indicate the b and y″ ions observed in ESI tandem mass spectra of the products of incubation with His_6_-VenL followed by treatment with GluC. The observed fragmentation pattern is shown for (A) VenL-modified VenA, (B) VenL-modified VenA-C34A, (C) VenL-modified VenA-C45A, (D) VenL-modified VenA-C32A, and (E) VenL-modified VenA-C50A. Dehydrated amino acids and cysteine residues in each analog are shown in blue and green, respectively. See Figure S3 for the MS/MS spectra. (F) The topology of (methyl)lanthionines in venezuelin. Magenta arrows indicate the position and direction of Lan/MeLan ring formation.

In the tandem mass spectra of the VenA, VenA-C34A, and VenA-C45A peptides, no fragmentation was observed in the C-terminal region (Dhb31–Ala51) except for a fragment ion resulting from cleavage between Cys50 and Ala51 (Figure 4A–C). This observation suggests that the processed VenA peptide contains overlapping cyclic structures between Dhb31 and Cys50; such cyclic structures are less susceptible to fragmentation than linear regions [8]. Additional ions were observed in the C-terminal region in the spectrum of VenL-processed VenA-C32A (Figure 4D), providing more insights into the ring pattern. Two segments were still protected from fragmentation (Cys34–Dhb39 and Cys45–Dha49), implying the formation of a MeLan between Cys34 and Dhb39 and a Lan between Cys45 and Dha49. The fragmentations that are observed around Dhb32 and Dhb43 in this mutant suggest that these two Dhb residues, whose cyclization partners would be Cys32 and Cys50, are not cyclized. Hence, disruption of a MeLan involving Cys32, which is mutated in VenA-C32A, appears to interfere with formation of another ring. Nonetheless, the fragmentation pattern limits the ring topology to two possibilities: Cys32–Dhb43 and Cys50–Dhb31, or Cys32–Dhb31 and Cys50–Dhb43, in addition to the two assigned rings (Cys34–Dhb39 and Cys45–Dha49). VenA-C50A was also treated with VenL and the product analyzed by ESI-Q/ToF MS (Figure 4E). The large number of fragmentations in the C-terminal region suggests that Cys45 is not cyclized by VenL when Cys50 is mutated. On the other hand, protection from fragmentation in the N-terminal region strongly supports ring formation between Cys32 and Dhb43. With three rings assigned, the final ring would have to form between Cys50 and Dhb31. Collectively, the only structure that can account for the regions that are protected from fragmentation in all VenA peptides tested is the ring topology shown in Figure 4F. Future NMR studies will be needed to further confirm this structure, but these will require much larger amounts of material than can currently be obtained.

The proposed ring pattern is not found in known lantibiotics and most closely resembles the globular structures of cinnamycin and the duramycins, also products of streptomycetes [14],[26]. Both cinnamycin/duramycin and the structure proposed here for venezuelin contain four overlapping rings with one particularly large ring structure encompassing 18 amino acids in cinnamycin/duramycin and 20 amino acids in venezuelin.

### In Vitro Production of Venezuelin

Given the difficulties encountered in detecting venezuelin production in both *S. venezuelae* and *S. lividans*, we sought to apply in vitro techniques. All lantibiotics characterized thus far require the removal of the N-terminal leader sequence of the modified precursor peptide to attain their active forms. Since the cluster does not contain an identifiable protease or protease domain that might provide insights, the site of protease cleavage is unknown. However, a peptide with sequence homology to VenA encoded in the genome of *Streptomyces clavuligerus* has an AlaPheAla sequence that is identical to the cleavage site found in the CinA precursor to the lantibiotic cinnamycin (Figure S4A). For cinnamycin, this cleavage site is thought to be recognized by a protease of the secretory machinery [14]. In VenA, this AlaPheAla-like sequence is a ProPheAla sequence spanning positions 27–29. Thus, several mutant VenA peptides were generated in which cleavage sites for commercial proteases were engineered such that after proteolysis the C-terminal product would be that predicted by a ProPheAla cleavage site (Figure S5A). Alternatively, GlyAla and AlaAla sequences are found in VenA that could be potential cleavage sites for a protease of the double-Gly type that are found in class II lantibiotics [27]. These sequences are located at positions 25–26 and 29–30 of VenA, respectively. To investigate these potential cleavage sites, a series of VenA analogues were generated in which either a Lys-C/trypsin or Factor Xa cleavage site was introduced (Figure S5A). All five mutant peptides were expressed and purified as His_6_-tagged peptides and incubated with His_6_-VenL resulting in the anticipated four dehydrations. Furthermore the products were devoid of free thiols as determined by IAA alkylation as described above. All VenL-processed peptides were then treated with the corresponding protease and the products were tested against *Micrococcus luteus* ATCC4698, *Lactococcus lactis* HP, and *Bacillus subtilis* LH45 using well-diffusion assays. These three strains are highly susceptible to a wide range of lantibiotics. However, no antimicrobial activity was detected (Figure S5B) under the conditions used.

## Discussion

**Figure 5:**
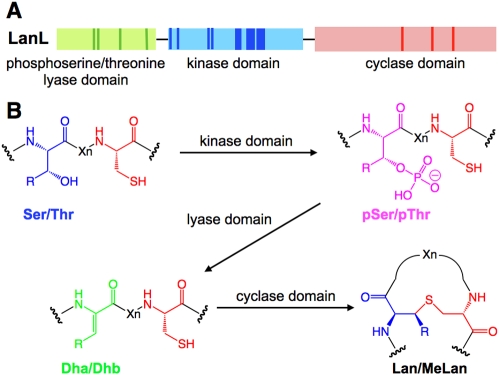
Posttranslational modification by LanL enzymes. (A) Illustration of the three catalytic domains constituting LanL enzymes. The positions of conserved residues important for catalysis are shown in darker colors. (B) Scheme of post-translational modification by LanL enzymes to install Lan/MeLan into the substrate LanA peptides. R = H or Me.

We describe a novel class of lanthionine synthetases consisting of three catalytic modules (Figure 5A) that install the thioether rings via phosphorylation of Ser/Thr residues by a kinase-like domain, elimination of the phosphate by a lyase domain, and cyclization by a LanC-like cyclase domain (Figure 5B). These enzymes appear to possess the same cyclization strategy as the previously discovered LanM and LanC proteins, retaining the conserved zinc binding site that is believed to activate the Cys residues for nucleophilic attack onto the dehydro amino acids [10]–[12]. However, the dehydration reaction is carried out in two separate domains that have no significant sequence homology with either the LanB or the LanM proteins (Figures S7, S8, and S9). The central part of LanL proteins (residues 230–487 in VenL) contains all 12 conserved subdomains of protein serine/threonine kinases [28]. Importantly, the highly conserved residues in these kinases are also well conserved in LanL proteins. On the other hand, the characteristic kinase motifs found in VenL are not conserved in either LanB or LanM enzymes (Figures S8 and S9B).

The N-terminal part (residues 1–163) of VenL has homology to members of the OspF protein family. Most importantly, Lys51, His53, Arg83, and Tyr108 in VenL align well with the residues that play essential roles in catalysis by OspF enzymes (Lys104, His106, Lys136, and Tyr158 in SpvC, an OspF family member from *Salmonella*). In the proposed catalytic mechanism of SpvC based on an X-ray structure [21], Lys104 and Tyr158 donate hydrogen bonds to the carbonyl oxygen of the target phosphothreonine to decrease the pK_a_ of its α proton. Lys136 is the catalytic base that abstracts the α proton triggering the β-elimination of phosphate, with His106 protonating the oxygen of the phosphate leaving group. An important difference between the OspF and LanL proteins is that the latter process several phosphorylated Ser/Thr residues present in one peptide, unlike the OspF family, which recognizes one specific phosphoThr in host MAPKs. The essential residues of the lyase domain are not found in LanB proteins (Figure S9), but sequence alignments with a series of LanM enzymes suggested that possibly some of the conserved residues in OspF might also be present at the N-termini of LanM enzymes (Figure S7A). To test the functional importance of these residues, mutants were generated in a representative LanM enzyme (lacticin 481 synthetase; LctM) in which the conserved residues that may be part of a putative lyase active site were mutated (Lys227Met, Tyr225Ala, and the double mutation Arg226Met/Lys227Met). When incubated with the LctA substrate, all three mutants had full dehydration activity (Figure S7B), strongly suggesting that these residues are not part of a lyase domain in LctM.

**Figure 6:**
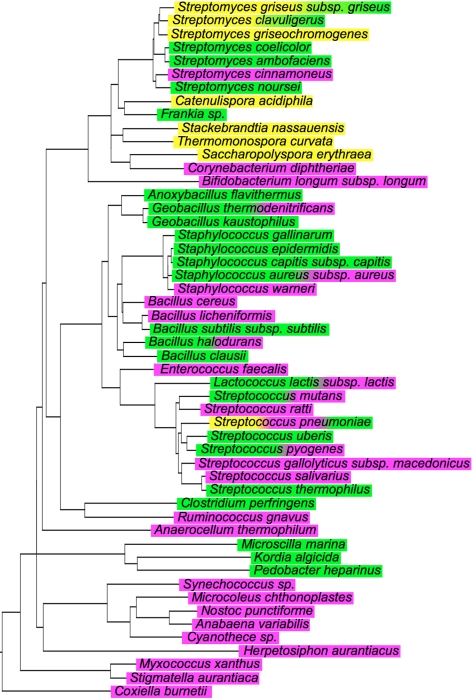
Phylogenetic tree of 16S rRNA of species having putative lantibiotic synthetase genes. Species with *lanL*, *lanB*, and *lanM* genes are highlighted in yellow, green, and magenta, respectively. The phylogenetic tree was constructed based on the Ribosomal Database Project [38]. For the bacterial strains used and gene accession numbers, see Tables S3 and S4.

The discovery of a third route for the synthesis of lanthionine-containing peptides illustrates the biological importance of these conformationally constrained peptides. Given the remarkable efficiency of forming polycyclic peptides from linear ribosomally produced peptides and their proven capability to recognize molecular targets with high affinities, it is perhaps not surprising that nature has evolved three distinct families of polypeptide sequences in order to access the reactive dehydro amino acids Dha and Dhb. A search of the available protein databases shows that the genes responsible for the three strategies for lanthionine biosynthesis are widespread, with some species containing two or all three pathways; e.g., LanL and LanB in *Streptomyces clavuligerus* and *Streptomyces griseus*, LanM and LanB in *Streptococcus pyogenes* and *Lactococcus lactis*, and all three pathways in *Streptococcus pneumoniae* (Figure 6). The wealth of genomic information that is now available shows that putative lantibiotic biosynthetic gene clusters are not restricted to Gram-positive organisms as long believed (see also [29]). Such gene clusters are also found in Gram-negative bacteria such as the proteobacterium *Myxococcus xanthus* and in cyanobacteria such as *Nostoc punctiforme*.

We propose that the VenL proteins evolved from stand alone protein Ser/Thr kinases and phosphoSer/Thr lyases. The OspF proteins likely evolved high substrate specificity from a promiscuous ancestor, whereas the LanL proteins maintained low substrate specificity. Interestingly, although LanM enzymes do not contain the characteristic motifs of Ser/Thr kinases or phosphoThr lyases (Figures S7 and S8), recent in vitro studies of a LanM demonstrated that the Ser/Thr residues in the precursor peptide are phosphorylated followed by phosphate elimination to generate the dehydro amino acids [30],[31]. Thus, while on the sequence level the dehydratases in the three classes of peptide dehydratases have no obvious homology, the chemical logic to carry out the dehydration reaction may well be similar.

Interestingly, all three classes of putative lantibiotic synthetases utilize the same cyclization strategy. As mentioned previously [3], the cyclization reaction is chemically not very demanding and takes place readily non-enzymatically. However, control over chemo- and regio-selectivity requires enzyme catalysis [32], suggesting that primordial lantibiotics may have been mixtures of non-enzymatically cyclized compounds that became enzyme-guided to enrich the biologically active isomers by acquisition of zinc-containing proteins that are used widespread in nature for thiol alkylation [33],[34].

Historically, lantibiotics have been discovered from antimicrobial screens, but as more and more genomes are sequenced, bioinformatic analyses are likely to become a major route to the discovery of new compounds and one that is not limited by the biological activity defined by the screen. Many of the lantibiotic-like gene clusters discovered by genome scanning may direct the production of peptides that do not have antibiotic activity, but that may act, for example, as signaling molecules. In fact, two lanthionine-containing peptides from streptomycetes with morphogenetic activities have already been reported [18],[35]. Moreover, given the extraordinary efficiency of producing conformationally constrained peptides by the dehydration/cyclization strategy, it is likely that compounds generated by genetic diversification will have a variety of biological activities. The name lantibiotics was introduced in 1988 as a shortcut for lanthionine-containing antibiotics [36]. We would like to suggest the name lantipeptides for compounds that by structure and biosynthetic strategy are clearly related to lantibiotics but that are not known to possess antimicrobial activity.

In summary, a new family of lanthionine synthetases termed LanL was discovered in *S. venezuelae* that contain a phosphoSer/Thr lyase domain, a kinase domain, and a cyclase domain. These novel enzymes provide unique new insights into the potential evolutionary origin and the mechanism of these remarkable catalysts. The approach taken here demonstrates that genome scanning, combined with in vitro enzymology, can be a potential strategy to reveal novel mechanistic and evolutionary insights. The genes encoding LanL proteins are widespread in nature, as are lantibiotic/lantipeptide biosynthetic gene clusters in general.

## Materials and Methods

### Materials

*S. venezuelae* ATCC10712 was maintained as previously described [37]. Methods for cloning and protein purification are provided in the Supporting Information (Text S1 and Table S2).

### Modification Assay of His_6_-VenA with His_6_-VenL Analogues

His_6_-VenA was incubated with His_6_-VenL, His_6_-VenL-ΔC, His_6_-VenL-ΔLC, or both His_6_-VenL-ΔLC and His_6_-VenL-ΔKC (final concentration: 2 µM each for His_6_-VenL and His_6_-VenL-ΔC, 4 µM each for His_6_-VenL-ΔLC and His_6_-VenL-ΔKC) in a reaction buffer that contained (final concentrations) 50 mM HEPES·Na buffer (pH 7.5), 10 mM MgCl_2_, 2.5 mM ATP, 1 mM TCEP, 25 µM His_6_-VenA, and 5% DMSO. The reactions with His_6_-VenL and His_6_-VenL-ΔC were incubated at 25°C for 3 h and the assays with His_6_-VenL-ΔLC or His_6_-VenL-ΔKC were incubated for 15 h. For mass spectrometric analysis, 10 µL of each reaction mixture was desalted using ZipTip_C18_ (Millipore), eluted with 1 µL of a 75% MeCN/25% water solution containing 0.1% TFA and saturated sinapinic acid, and spotted onto the target plate for analysis by using a Voyager-DE-STR mass spectrometer (Applied Biosystems).

### IAA Modification Assay of Modified His_6_-VenA

After incubation of His_6_-VenA with His_6_-VenL in 20 µL of modification assay buffer, 6 µL of IAA reaction buffer (330 mM Tris-HCl buffer pH 8.5, 33 mM IAA, 6.7 mM TCEP) was added. The solution was incubated at 25°C for 15 h, desalted using ZipTip_C18_, and subjected to MALDI-ToF MS.

### LC-ESI-Q/ToF MS Analysis of Cyclized VenA Analogs

After treatment of each His_6_-VenA analog with His_6_-VenL, 0.3 U of endoproteinase Glu-C (Fluka) was added to 20 µL of assay solution. The solution was further incubated at 25°C for 18 h. An aliquot of 10 µL of the resulting sample was fractionated on an Acquity UPLC (Waters) equipped with a C8 column (100 mm×1 mm) using a gradient of 3%–97% B over 12 min (A =  water containing 0.1% formic acid, B =  methanol containing 0.1% formic acid), and directly subjected to ESI-Q/ToF MS (Synapt MS system, Waters). Nitrogen was used as cone gas (150 L/min) and desolvation gas (600 L/min). The capillary voltage was set to 3.5 kV. The ionization source and desolvation gas were heated to 120°C and 300°C, respectively. The ions having m/z = 1,104.34 (trivalent ion of VenA) or 1,093.65 (trivalent ions for the VenA mutants) were fragmented with a trap collision energy of 20–40 V. The acquired spectrum was converted to a deconvoluted spectrum by using the MaxEnt3 program (Waters).

## Supporting Information

Figure S1 **SDS-PAGE analysis of the proteins used in this study.** Lane 1, BioRad low range molecular weight standards. Lane 2, VenL full length protein (Calculated M.W.: 103 kDa); lane 3, VenL-ΔC (VenL truncated protein, 1–513 aa, Calculated M.W.: 57 kDa); lane 4, VenL-ΔKC (1–212 aa, Calculated M.W.: 25 kDa); lane 5, VenL-ΔLC (kinase domain, 201–513 aa, Calculated M.W.: 35 kDa); lane 6, Bio-Rad prestained SDS-PAGE standards, broad range.
(0.43 MB TIF)

Figure S2 **MALDI-ToF MS analysis of IAA modification assay of His_6_-VenA.** Mass spectra of (A) His_6_-VenA without IAA treatment, (B) His_6_-VenA after IAA treatment, (C) His_6_-VenA after incubation with His_6_-VenL followed by IAA treatment, and (D) His_6_-VenA after incubation with His_6_-VenL-ΔC followed by IAA treatment are shown.
(0.22 MB TIF)

Figure S3 **Tandem mass spectra of VenA analogs modified by VenL.** ESI-Q/ToF MS spectra of (A) VenA, (B) VenA-C32A, (C) VenA-C34A, (D) VenA-C45A, and (E) VenA-C50A after VenL treatment followed by Glu-C cleavage are shown. b and y″ ions are marked in the spectra. “i(XXXXX)” labels indicate the ions corresponding to internal peptide fragments resulting from two fragmentations. Asterisks indicate the peaks originating from the non-fragmented parent peptide, such as [M+H]^+^ and [M+2H]^2+^.
(1.37 MB TIF)

Figure S4 **Sequence alignments of VenA and its homologue, SclA.** All lantibiotics known to date require the removal of the N-terminal leader sequence of the modified precursor peptide to attain their active forms. Since venezuelin production by *S. venezuelae* has not yet been detected and since the cluster does not contain a protease that might provide insights, the site of protease cleavage is unknown. However, a peptide with sequence homology to VenA encoded in the genome of *Streptomyces clavuligerus* has an AFA sequence (panel A). Identical and similar residues are highlighted in yellow and cyan, respectively. The same AFA sequence motif has been predicted to be the recognition site for removal of the leader peptide of cinnamycin by Type I signal peptidases of the general secretory pathway [14]. In VenA, the homologous sequence is PFA^29^–A^30^, and we infer that the VenL-modified VenA is likely cleaved between Ala29 and Ala30 upon secretion (thick arrow). Taken together with the results of tandem mass spectrometry of the VenA derivatives, we propose the structure of venezuelin shown in Figure S4B. Thin arrows indicate the proposed Lan/MeLan ring formation in venezuelin. Attempts to detect production of the lanthionine-containing peptide of *Streptomyces clavuligerus* were unsuccessful.
(0.44 MB TIF)

Figure S5 **Bioactivity assay of venezuelin analogs prepared in vitro.** (A) Sequences of VenA mutants constructed to engineer protease recognition sites (shown in red, K for trypsin/LysC cleavage; IEGR for Factor Xa/trypsin cleavage) at various potential leader peptide cleavage sites. (B) Antimicrobial activity assay of the VenA mutants processed by VenL and subsequently treated with protease. The products were tested against *B. subtilis* LH45, *M. luteus* ATCC4698, and *L. lactis* HP. Spot 1, DMSO (negative control); spot 2, VenL and trypsin (negative control); spots 3–8, various amounts of VenA mutants modified by VenL and treated with trypsin; spot 9, haloduracin (positive control). None of the venezuelin analogs showed antimicrobial activity under these conditions. The very faint zones seen in all cases (but most obviously for *L. lactis* HP) are attributed to DMSO (negative control in spot 1), which was required because of the very poor aqueous solubility of VenA and its processed derivatives.
(1.71 MB TIF)

Figure S6 **Sequence alignments of LanL family members.** For more information on the LanL sequences, see Table S1. For more information on the strains used to generate the figure, see Tables S2 and S3.
(7.54 MB TIF)

Figure S7 **LanM enzymes do not contain the lyase domain found in the LanL family.** (A) Sequence alignment between 10 LanL proteins and four selected LanM proteins. The alignments suggest that the lyase domain found in LanL proteins is not present in LanM proteins since the catalytic residues involved in the lyase activity of the OspF family and conserved in the LanL family (red stars) are not conserved in LanM. However, some weak sequence similarity between the lyase domains of the LanL family and LanM enzymes is shown around residue 149 of VenL (red bar); no significant homology was observed in other regions. (B) Substrate modification assays with VenL-ΔC, LctM, and LctM mutants, in which the residues in the putative lyase domains of LanLs and LanMs were mutated in lacticin 481 synthetase (LctM). All mutants at these residues (Y225A, K227M, R226M/K227M) showed dehydration activity with the LctA substrate peptide similar to that of the wild-type LctM, which is shown for comparison. These results demonstrate that the region with weak homology does not play an important role in the dehydration activity of LctM.
(2.95 MB TIF)

Figure S8 **Sequence alignments of Ser/Thr protein kinases and the N-termini of selected LanM proteins.** The conserved residues that provide the catalytic loop and ATP binding pocket in the Ser/Thr kinases and LanL proteins are highlighted in cyan and are not conserved in LanM proteins. The few residues in LanM that do exhibit similarity to the conserved residues in Ser/Thr kinases are highlighted in green.
(4.53 MB TIF)

Figure S9 **Sequence alignment of LanB proteins with LanL proteins and with Ser/Thr protein kinases.** (A) Alignment of the lyase domains of the LanL family with LanB enzymes. The alignments suggest that the lyase domain is not present in LanB proteins since the essential catalytic residues in this domain, determined by studies on the OspF family of proteins (red stars), are not conserved in LanB. (B) Sequence alignments between protein kinases and LanB proteins. The conserved residues that provide the catalytic loop and ATP binding pocket in protein kinases are highlighted in cyan and are not conserved in LanB proteins. The residues in LanB that exhibit similarity to the conserved amino acids in the catalytic loop/ATP binding pocket of protein kinases are highlighted in green.
(7.53 MB TIF)

Table S1 **Putative LanL proteins found in the databases and the organisms of origin.**
(0.28 MB TIF)

Table S2 **Oligonucleotide primers used in this study.** ^a^ Restriction sites are underlined. ^b^ Mutant nucleotides are highlighted in bold.
(0.76 MB TIF)

Table S3 **List of bacteria containing LanB, LanM, and LanL genes.** The genes that were used to construct Figure 6 and their accession numbers are shown. The list is not meant to be comprehensive (for instance some of the known lantibiotic producers were not included) but meant to illustrate the general distribution of these genes.
(0.02 MB PDF)

Table S4 **Species and strains with genes for more than one pathway to lanthionine-containing peptides.** Figure 6 in the main text shows species with genes for more than one pathway to lanthionine-containing peptides. In some cases, these genes are found in one strain; in other cases, the genes are found in different strains. For accession numbers, see Table S3.
(0.01 MB PDF)

Text S1 **Molecular biology procedures, antimicrobial assays, and enzyme expression and purification protocols.** Description of procedures used to construct the plasmids VenL/pET28, VenL-ΔC/pET28, VenL-ΔLC/pET28, VenL-ΔKC/pET28, and MBP-VenA/pET28. Also provided are the expression and purification protocols for these proteins and the methods used for site-directed mutagenesis of *venA*. Construction of the Δ*venL*, Δ*venA*, and *ermE*p*::*venL* derivatives of *S. venezuelae* are also described as well as antimicrobial assays with venezuelin and its analogs.
(0.09 MB PDF)

## Abbreviations

ATP: adenosine triphosphate
Dha: dehydroalanine
Dhb: *Z*-dehydrobutyrine
ESI-Q/ToF MS: electron spray ionization-quadrupole/time-of-flight mass spectrometry
His_6_: hexahistidine tag
IAA: iodoacetamide
IMAC: immobilized metal affinity chromatography
Lan: lanthionine
MALDI-ToF MS: matrix-assisted laser desorption/ionization time-of-flight mass spectrometry
MBP: maltose-binding protein
MeLan: methyllanthionine
TCEP: tris(2-carboxyethyl)phosphine
