# Supplementary figures and images for: Discovery of Unique Lanthionine Synthetases Reveals New Mechanistic and Evolutionary Insights
Source: PLoS Biol. 2010 Mar 23;8(3):e1000339. doi: 10.1371/journal.pbio.1000339 (PMC2843593; doi:10.1371/journal.pbio.1000339)

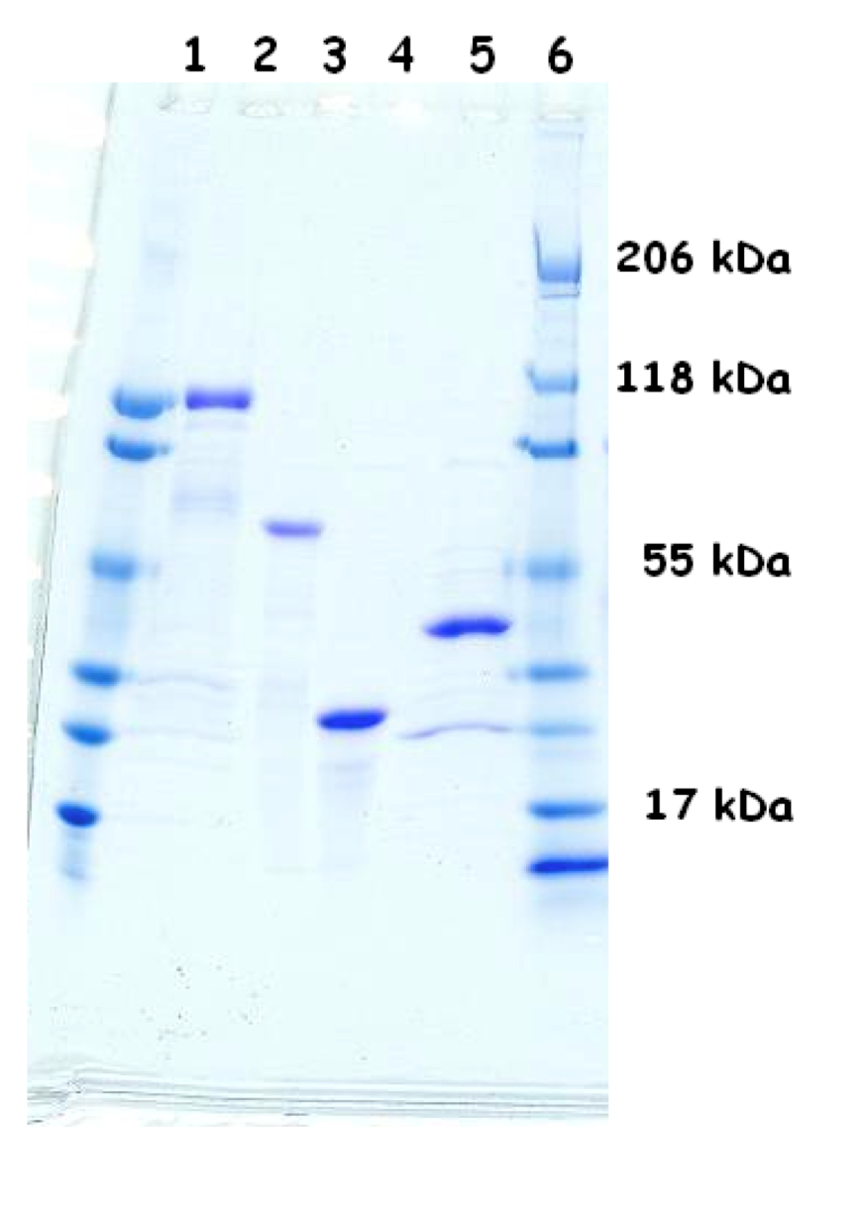

Supplement: Figure S1 — SDS-PAGE analysis of the proteins used in this study. Lane 1, BioRad low range molecular weight standards. Lane 2, VenL full length protein (Calculated M.W.: 103 kDa); lane 3, VenL-ΔC (VenL truncated protein, 1–513 aa, Calculated M.W.: 57 kDa); lane 4, VenL-ΔKC (1–212 aa, Calculated M.W.: 25 kDa); lane 5, VenL-ΔLC (kinase domain, 201–513 aa, Calculated M.W.: 35 kDa); lane 6, Bio-Rad prestained SDS-PAGE standards, broad range. (0.43 MB TIF) [file pbio.1000339.s001.tif]

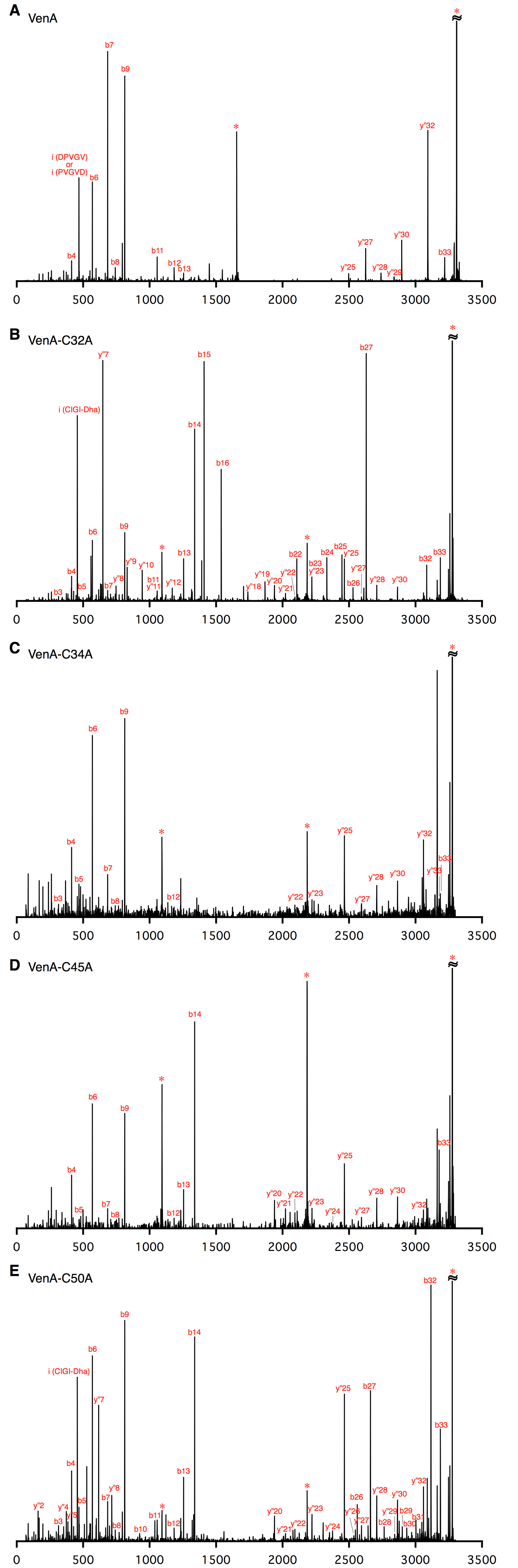

Supplement: Figure S3 — Tandem mass spectra of VenA analogs modified by VenL. ESI-Q/ToF MS spectra of (A) VenA, (B) VenA-C32A, (C) VenA-C34A, (D) VenA-C45A, and (E) VenA-C50A after VenL treatment followed by Glu-C cleavage are shown. b and y″ ions are marked in the spectra. “i(XXXXX)” labels indicate the ions corresponding to internal peptide fragments resulting from two fragmentations. Asterisks indicate the peaks originating from the non-fragmented parent peptide, such as [M+H]+ and [M+2H]2+. (1.37 MB TIF) [file pbio.1000339.s003.tif]

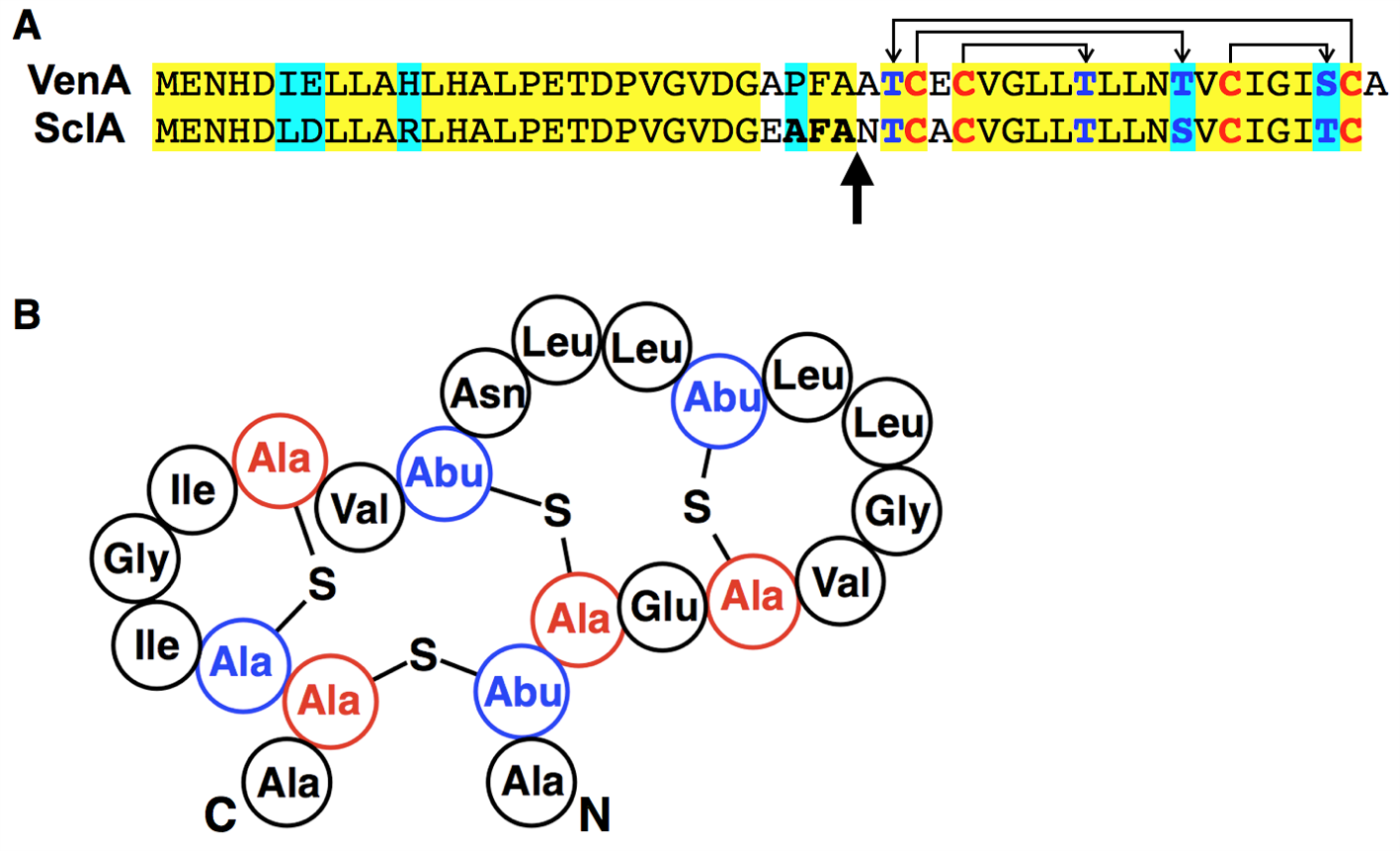

Supplement: Figure S4 — Sequence alignments of VenA and its homologue, SclA. All lantibiotics known to date require the removal of the N-terminal leader sequence of the modified precursor peptide to attain their active forms. Since venezuelin production by S. venezuelae has not yet been detected and since the cluster does not contain a protease that might provide insights, the site of protease cleavage is unknown. However, a peptide with sequence homology to VenA encoded in the genome of Streptomyces clavuligerus has an AFA sequence (panel A). Identical and similar residues are highlighted in yellow and cyan, respectively. The same AFA sequence motif has been predicted to be the recognition site for removal of the leader peptide of cinnamycin by Type I signal peptidases of the general secretory pathway [14]. In VenA, the homologous sequence is PFA29–A30, and we infer that the VenL-modified VenA is likely cleaved between Ala29 and Ala30 upon secretion (thick arrow). Taken together with the results of tandem mass spectrometry of the VenA derivatives, we propose the structure of venezuelin shown in Figure S4B. Thin arrows indicate the proposed Lan/MeLan ring formation in venezuelin. Attempts to detect production of the lanthionine-containing peptide of Streptomyces clavuligerus were unsuccessful. (0.44 MB TIF) [file pbio.1000339.s004.tif]

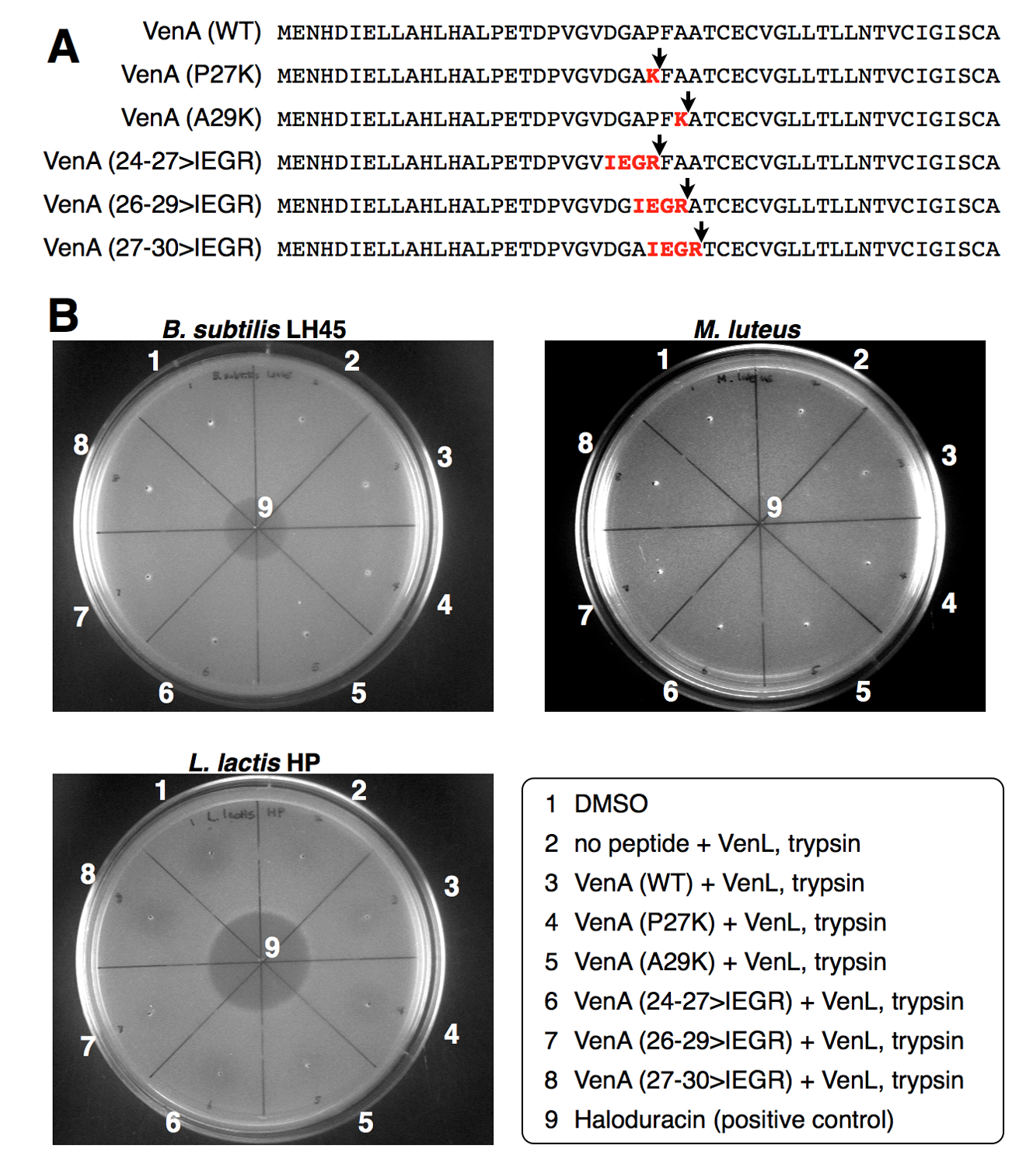

Supplement: Figure S5 — Bioactivity assay of venezuelin analogs prepared in vitro. (A) Sequences of VenA mutants constructed to engineer protease recognition sites (shown in red, K for trypsin/LysC cleavage; IEGR for Factor Xa/trypsin cleavage) at various potential leader peptide cleavage sites. (B) Antimicrobial activity assay of the VenA mutants processed by VenL and subsequently treated with protease. The products were tested against B. subtilis LH45, M. luteus ATCC4698, and L. lactis HP. Spot 1, DMSO (negative control); spot 2, VenL and trypsin (negative control); spots 3–8, various amounts of VenA mutants modified by VenL and treated with trypsin; spot 9, haloduracin (positive control). None of the venezuelin analogs showed antimicrobial activity under these conditions. The very faint zones seen in all cases (but most obviously for L. lactis HP) are attributed to DMSO (negative control in spot 1), which was required because of the very poor aqueous solubility of VenA and its processed derivatives. (1.71 MB TIF) [file pbio.1000339.s005.tif]

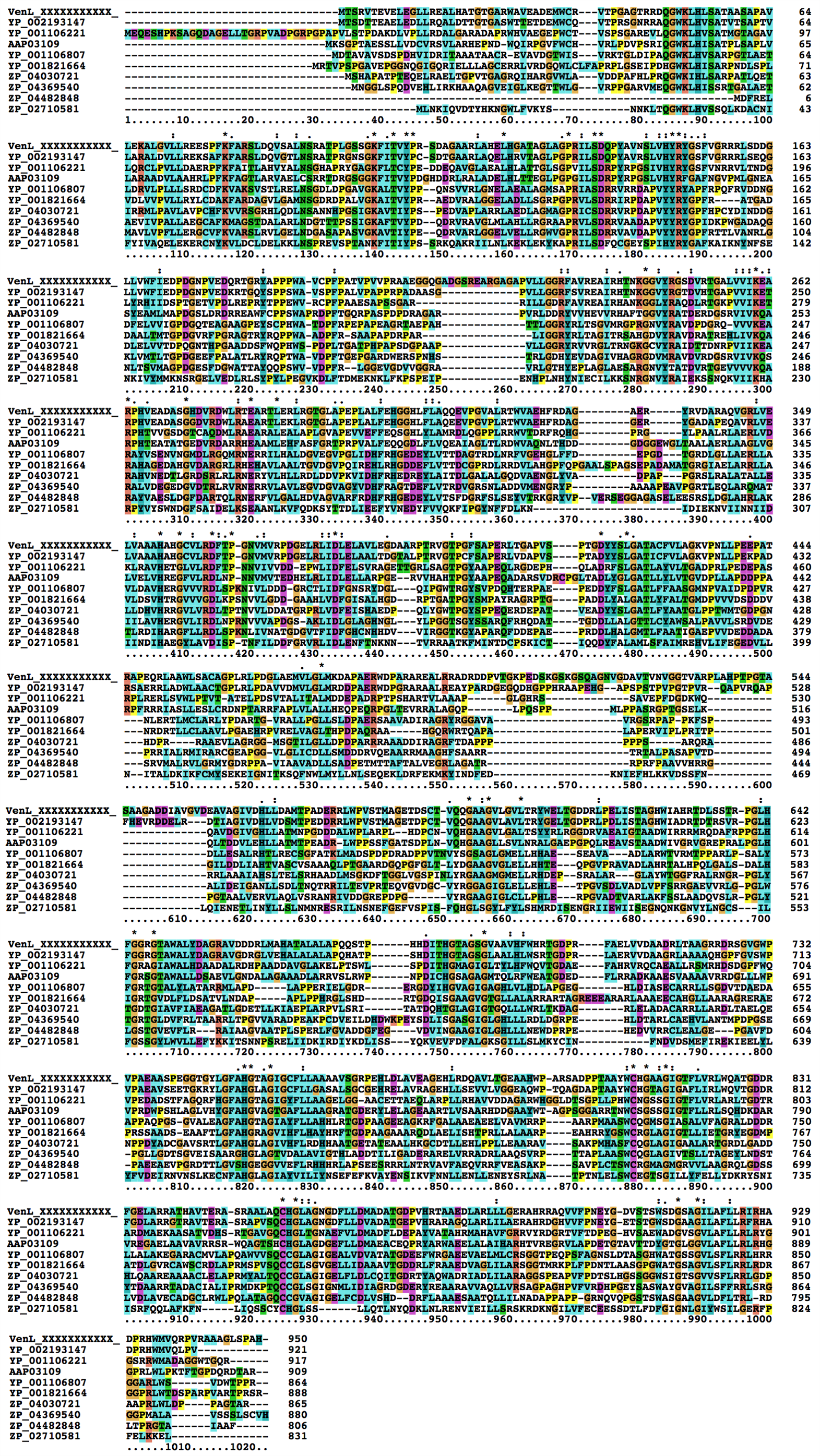

Supplement: Figure S6 — Sequence alignments of LanL family members. For more information on the LanL sequences, see Table S1. For more information on the strains used to generate the figure, see Tables S2 and S3. (7.54 MB TIF) [file pbio.1000339.s006.tif]

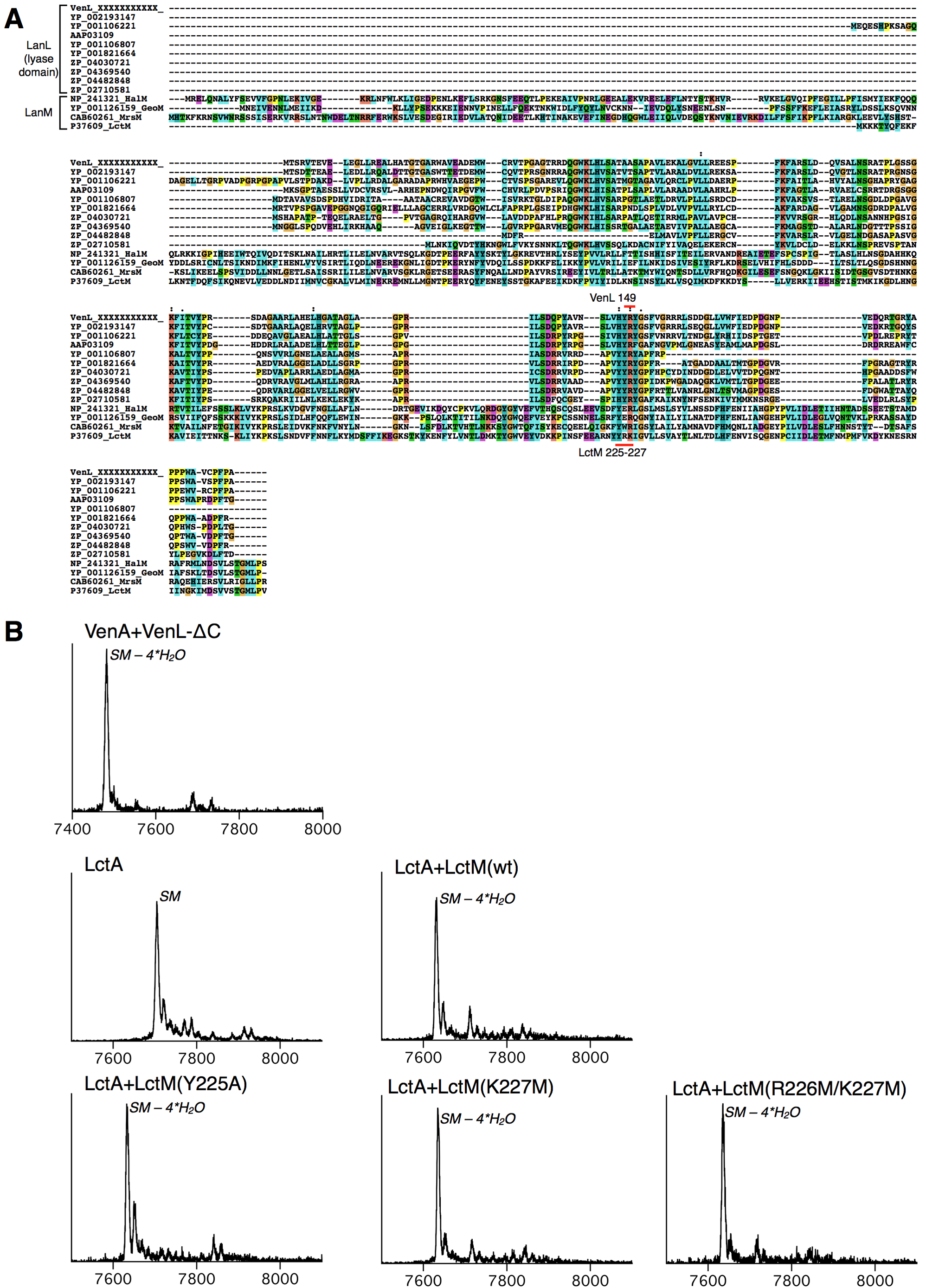

Supplement: Figure S7 — LanM enzymes do not contain the lyase domain found in the LanL family. (A) Sequence alignment between 10 LanL proteins and four selected LanM proteins. The alignments suggest that the lyase domain found in LanL proteins is not present in LanM proteins since the catalytic residues involved in the lyase activity of the OspF family and conserved in the LanL family (red stars) are not conserved in LanM. However, some weak sequence similarity between the lyase domains of the LanL family and LanM enzymes is shown around residue 149 of VenL (red bar); no significant homology was observed in other regions. (B) Substrate modification assays with VenL-ΔC, LctM, and LctM mutants, in which the residues in the putative lyase domains of LanLs and LanMs were mutated in lacticin 481 synthetase (LctM). All mutants at these residues (Y225A, K227M, R226M/K227M) showed dehydration activity with the LctA substrate peptide similar to that of the wild-type LctM, which is shown for comparison. These results demonstrate that the region with weak homology does not play an important role in the dehydration activity of LctM. (2.95 MB TIF) [file pbio.1000339.s007.tif]

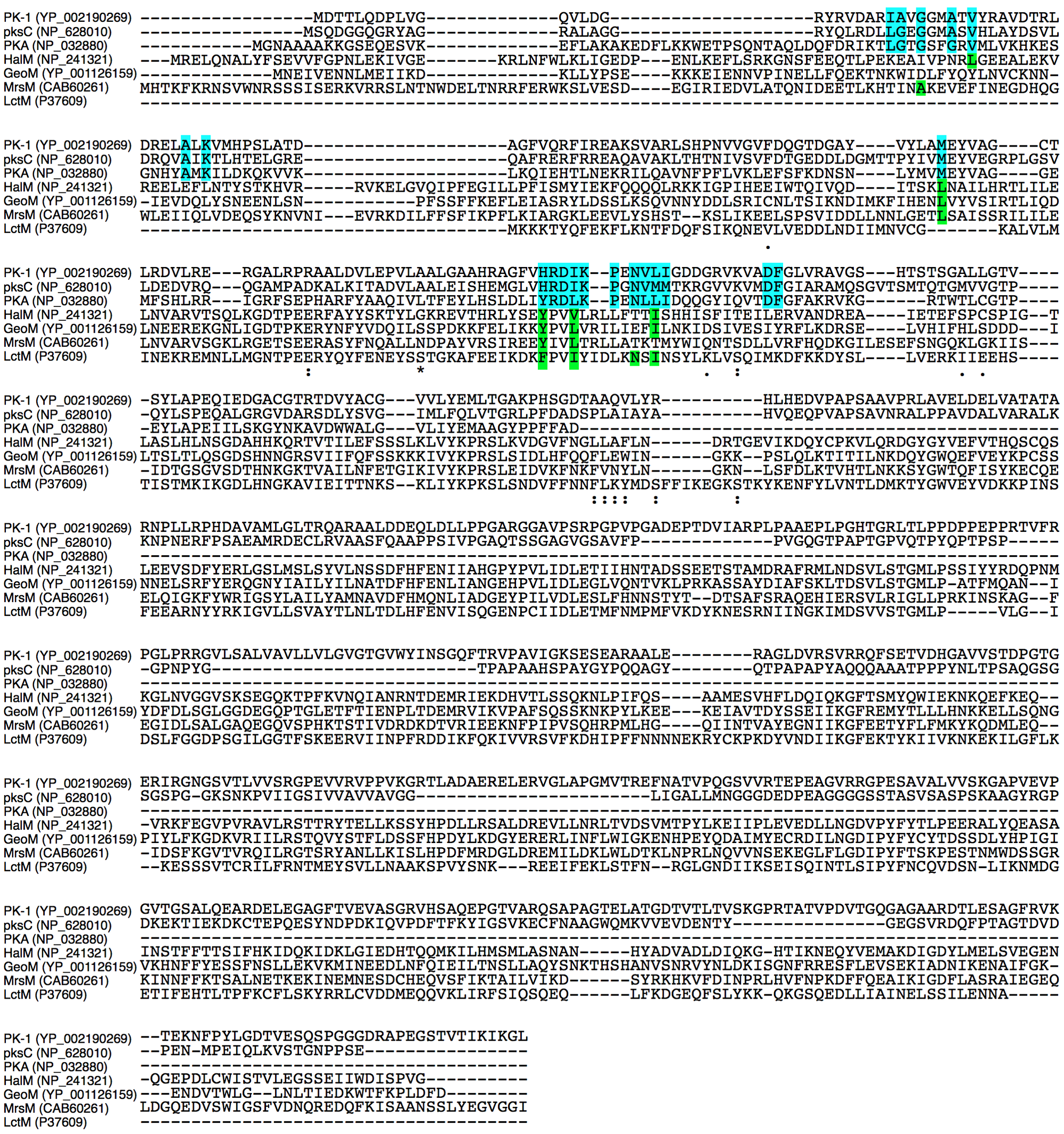

Supplement: Figure S8 — Sequence alignments of Ser/Thr protein kinases and the N-termini of selected LanM proteins. The conserved residues that provide the catalytic loop and ATP binding pocket in the Ser/Thr kinases and LanL proteins are highlighted in cyan and are not conserved in LanM proteins. The few residues in LanM that do exhibit similarity to the conserved residues in Ser/Thr kinases are highlighted in green. (4.53 MB TIF) [file pbio.1000339.s008.tif]

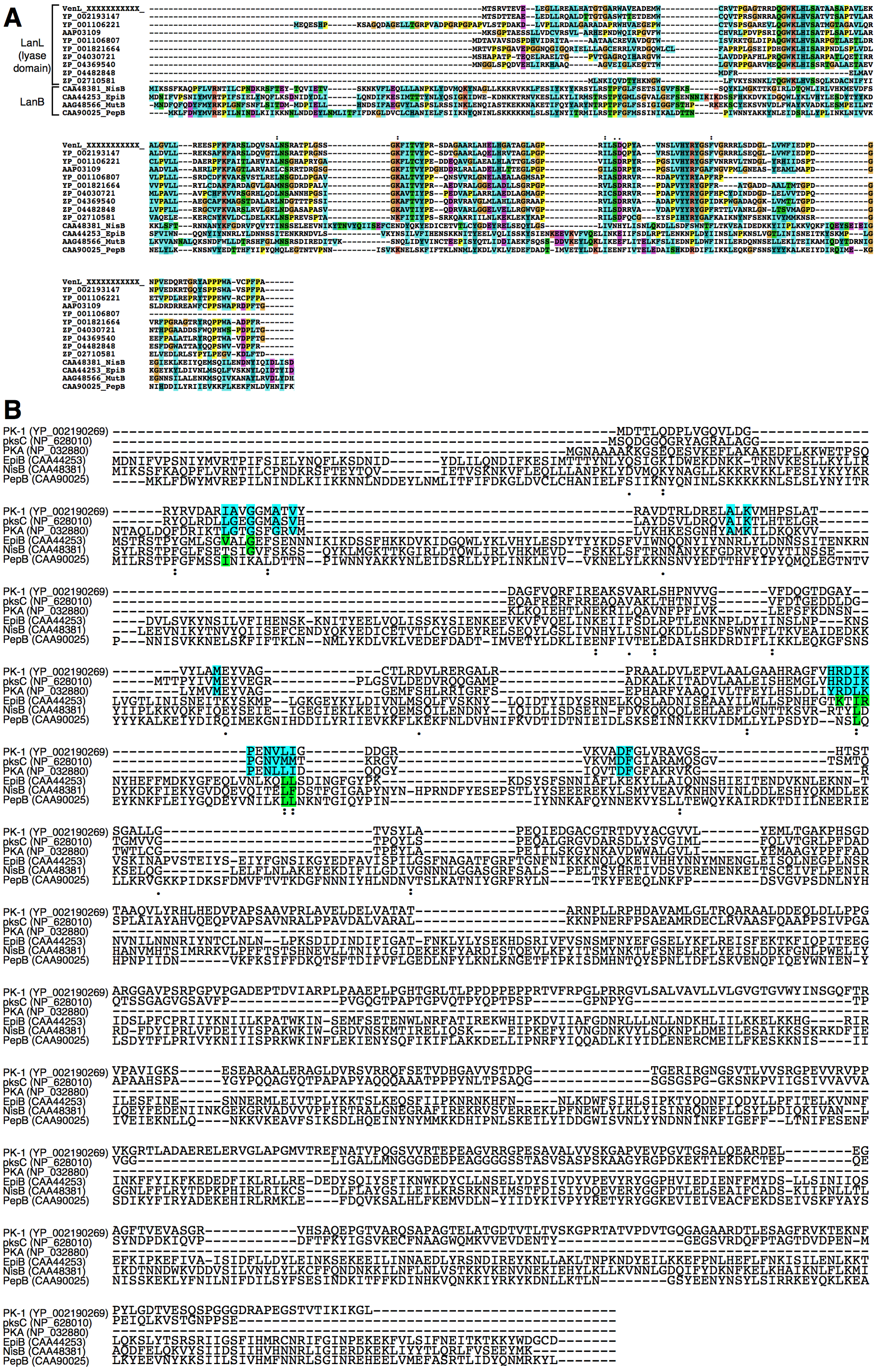

Supplement: Figure S9 — Sequence alignment of LanB proteins with LanL proteins and with Ser/Thr protein kinases. (A) Alignment of the lyase domains of the LanL family with LanB enzymes. The alignments suggest that the lyase domain is not present in LanB proteins since the essential catalytic residues in this domain, determined by studies on the OspF family of proteins (red stars), are not conserved in LanB. (B) Sequence alignments between protein kinases and LanB proteins. The conserved residues that provide the catalytic loop and ATP binding pocket in protein kinases are highlighted in cyan and are not conserved in LanB proteins. The residues in LanB that exhibit similarity to the conserved amino acids in the catalytic loop/ATP binding pocket of protein kinases are highlighted in green. (7.53 MB TIF) [file pbio.1000339.s009.tif]

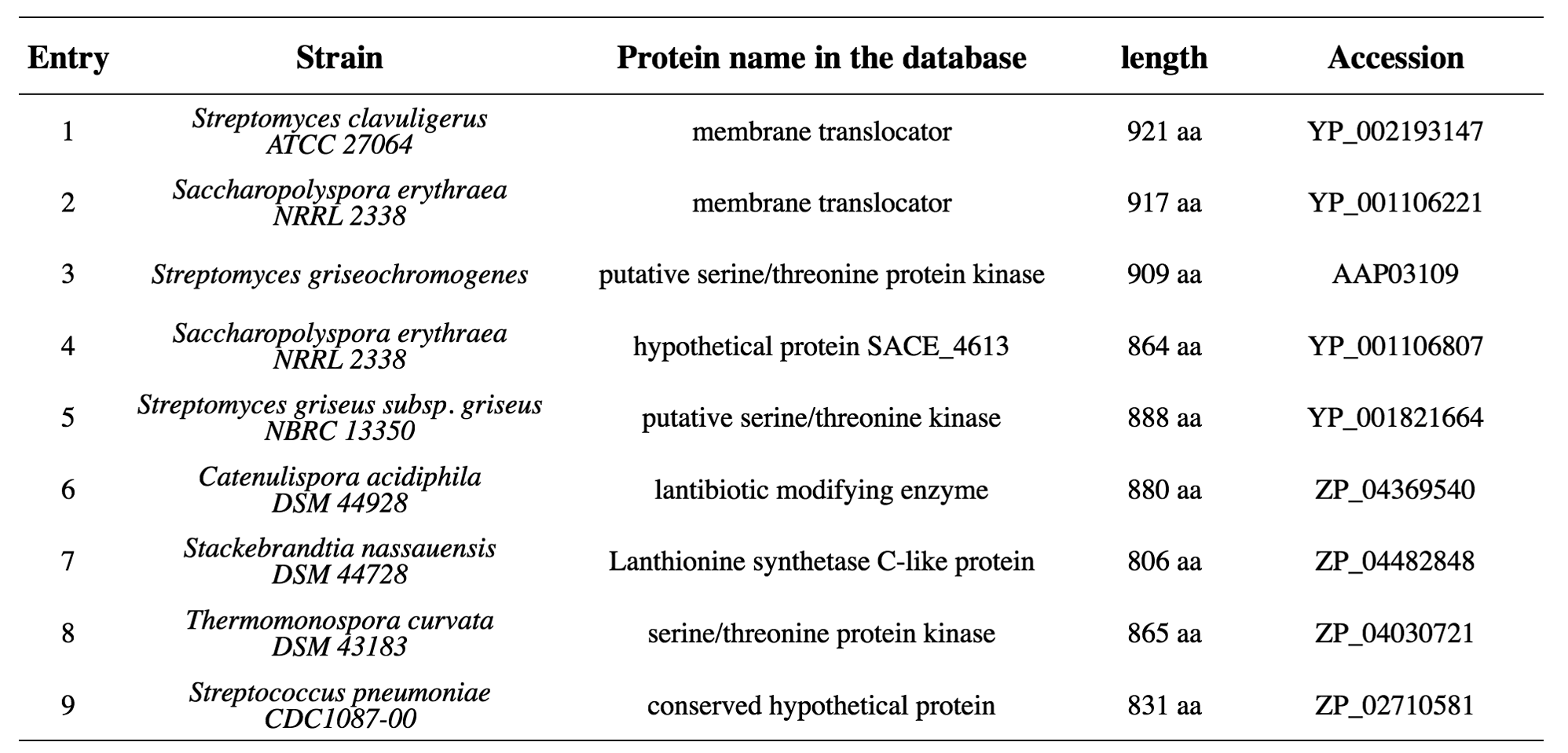

Supplement: Table S1 — Putative LanL proteins found in the databases and the organisms of origin. (0.28 MB TIF) [file pbio.1000339.s010.tif]

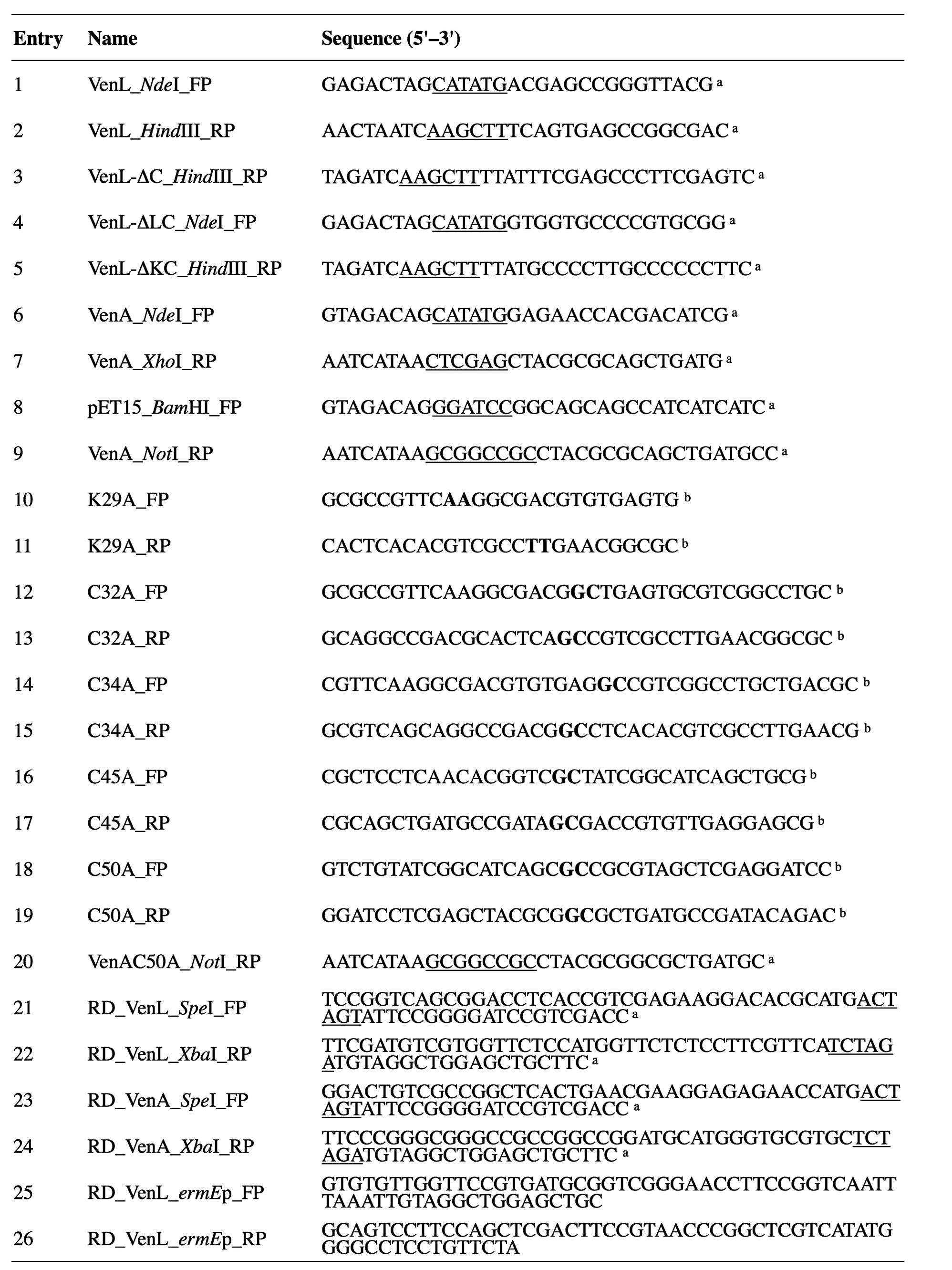

Supplement: Table S2 — Oligonucleotide primers used in this study. a Restriction sites are underlined. b Mutant nucleotides are highlighted in bold. (0.76 MB TIF) [file pbio.1000339.s011.tif]
