# Supplementary material for: Discovery of Unique Lanthionine Synthetases Reveals New Mechanistic and Evolutionary Insights
Source: PLoS Biol. 2010 Mar 23;8(3):e1000339. doi: 10.1371/journal.pbio.1000339 (PMC2843593; doi:10.1371/journal.pbio.1000339)
Supplement: Table S3 — List of bacteria containing LanB, LanM, and LanL genes. The genes that were used to construct Figure 6 and their accession numbers are shown. The list is not meant to be comprehensive (for instance some of the known lantibiotic producers were not included) but meant to illustrate the general distribution of these genes. (0.02 MB PDF) [file pbio.1000339.s012.pdf]

Bacteria with fully sequenced genomes containing LanB genes used for Figure 6.

Geobacillus thermodenitrificans NG80-2;	YP_001124395
Streptococcus pyogenes MGAS10750;	YP_602452
Streptococcus pyogenes MGAS9429;	YP_596657
Streptococcus pyogenes MGAS10270;	YP_598531
Streptococcus pyogenes MGAS6180;	YP_280252
Geobacillus sp. G11MC16;	ZP_03149197
Clostridium perfringens CPE str. F4969;	ZP_02640727
Bacillus clausii KSM-K16;	YP_177053
Geobacillus kaustophilus HTA426;	YP_146153
Anoxybacillus flavithermus WK1;	YP_002316782
Staphylococcus capitis SK14;	ZP_03613908
Streptococcus pneumoniae CGSP14;	YP_001834862
Streptomyces griseus subsp. griseus NBRC 13350;	YP_001827459
Streptococcus thermophilus LMG 18311;	YP_138642
Streptococcus thermophilus CNRZ1066;	YP_140529
Staphylococcus aureus subsp. aureus USA300_FPR3757;	YP_494457
Staphylococcus aureus subsp. aureus COL;	YP_186704
Staphylococcus aureus subsp. aureus MW2;	NP_646581
Staphylococcus aureus subsp. aureus MSSA476;	YP_043861
Streptomyces sp. Mg1;	ZP_04997226;<br>ZP_05001469;<br>ZP_04999931
Streptomyces coelicolor A3(2);	NP_630996;<br>NP_624599
Microscilla marina ATCC 23134;	ZP_01693365;<br>ZP_01690534;<br>ZP_01689156;<br>ZP_01694962
Frankia sp. EuI1c;	ZP_06240729
Frankia sp. EAN1pec;	YP_001505690;<br>YP_001507128
Frankia sp. CcI3;	YP_482401;<br>YP_480236;<br>YP_480926;<br>YP_481136
Kordia algicida OT-1;	ZP_02161440
Streptomyces clavuligerus ATCC 27064;	ZP_05005990;<br>ZP_05003917
Pedobacter heparinus DSM 2366;	YP_003090841

Other LanB genes used for Figure 6 that are from partially sequenced genomes and from bacteria producing known lantibiotics (shown in parentheses)

Streptococcus pyogenes MGA2096;	YP_600477
Staphylococcus gallinarum 3928 (gallidermin)	GdmB ABC94903
Streptomyces noursei ATCC 11455;	AAX37279

Streptomyces ambofaciens ATCC 23877;	CAJ88053
Lactococcus lactis 6F3; (nisin)	NisB CAA48381|
Streptococcus uberis strain 42; (nisin U)	NsuB ABA00879
Bacillus subtilis ATCC 6633; (subtilin)	SpaB AAB91586
Streptococcus mutans CH43/UA140; (mutacin I)	MutB AAG48566
Staphylococcus epidermidis; Tu 3298 (epidermin)	EpiB CAA44253;
Staphylococcus epidermidis strain 5	PepB CAA90025

Bacteria with fully sequenced genomes containing LanM genes (known lantibiotics in parentheses)

Anaerocellum thermophilum DSM 6725;	YP_002572981
Bacillus cereus ATCC 4342;	ZP_04287168; ZP_04287189
Bacillus cereus Rock1-3;	ZP_04248715
Bacillus cereus Q1;	YP_002532646
Bacillus cereus F65185;	ZP_04206897; ZP_04204173
Bacillus cereus 172560W;	ZP_04309311
Bacillus cereus Rock3-29;	ZP_04231453
Bacillus cereus AH621;	ZP_04298130
Bacillus cereus AH1272;	ZP_04183889
Bacillus licheniformis ATCC 14580/DSM-13; (lichenicidin)	YP_093632; YP_081203; YP_081205
Herpetosiphon tiacus ATCC 23779;	YP_001544639; YP_001546502
Bacillus halodurans C-125; (haloduracin)	NP_241321; NP_241318
Enterococcus faecalis ATCC 4200;	ZP_05474920
Geobacillus sp. G11MC16;	ZP_03149642
Cyanothece sp. PCC 7425;	YP_002485891; YP_002483742; YP_002484655; YP_002483601
Cyanothece sp. PCC 8802;	YP_003137732
Cyanothece sp. PCC 8801;	YP_002372173
Microcoleus chthonoplastes PCC 7420;	ZP_05025883
Geobacillus thermodenitrificans NG80-2;	YP_001126159
Streptococcus pyogenes MGAS315;	NP_665455
Streptococcus pyogenes MGAS10750;	YP_603221
Nostoc punctiforme PCC 73102;	YP_001866693; YP_001869999; YP_001868329; YP_001866601
Nostoc sp. PCC 7120;	NP_486065
Anabaena variabilis ATCC 29413;	YP_320138
Streptococcus pneumoniae ATCC 700669;	YP_002511204
Streptococcus pneumoniae SP23-BS72;	ZP_01834975
Streptococcus pneumoniae R6;	NP_359359

Streptococcus pneumoniae CDC0288-04;	ZP_02716217
Streptococcus pneumoniae TIGR4;	NP_346378
Streptococcus pneumoniae ATCC 700669;	YP_002511832
Ruminococcus sp. 5_1_39B_FAA;	ZP_04857854
Ruminococcus flavefaciens FD-1;	ZP_06143781; ZP_06143782
Synechococcus sp. RS9916;	ZP_01470939
Myxococcus xanthus DK 1622;	YP_631068; YP_634512
Stigmatella aurantiaca DW4/3-1;	ZP_01460524
Staphylococcus warneri ISK-1; (nukacin ISK-1)	NP_940773
Streptococcus mutans NN2025	BAH87352;
Coxiella burnetii Dugway 5J108-111;	YP_001424603
Coxiella burnetii 'MSU Goat Q177';	ZP_01946732
Coxiella burnetii CbuG_Q212;	YP_002303280

Other LanM genes used for Figure 6 that are from partially sequenced genomes or from bacterial strains producing known lantibiotics (given in parentheses)

Streptomyces cinnamoneus;	CAD60521
Streptococcus macedonicus; (macedocin)	McdM ABI30229
Streptococcus mutans T8; (mutacin II)	MutM AAC38145;
Streptococcus mutans (mutacin K8)	MukM ABK59358;
Streptococcus mutans GS5 (Smb)	BAD72771
Streptococcus pneumoniae CDC1873-00;	CylM ZP_02709201
Streptococcus ratti; (BHT)	BhtM1 AAZ76597
Streptococcus pyogenes FF22; (Streptococcin A-FF22)	ScnM AAB92602
Bacillus sp. HIL-Y85/54728; (mersacidin)	MrsM CAB60261
Lactococcus lactis subsp. lactis C2102; (lacticin 481)	LctM P37609
Ruminococcus gnavus E1 (ruminococcin)	RumM CAB93674; AAK73192
Streptococcus salivarius K12; (salivaricins)	SivM ABI54435; SivM ACX68644; SboM ABI63640; SalM ABI63629

Bacterial strains containing LanL genes

Streptomyces clavuligerus ATCC 27064;	YP_002193147
Saccharopolyspora erythraea NRRL 2338;	YP_001106221; YP_001106807
Streptomyces griseus subsp. griseus NBRC 13350;	YP_001821664
Catenulispora acidiphila DSM 44928;	ZP_04369540
Stackebrandtia nassauensis DSM 44728;	ZP_04482848
Thermomonospora curvata DSM 43183;	ZP_04030721
Streptococcus pneumoniae CDC1087-00;	ZP_02710581
Streptomyces griseochromogenes (partial genome sequenced)	AAP03109
