# Supplementary material for: Discovery of Unique Lanthionine Synthetases Reveals New Mechanistic and Evolutionary Insights
Source: PLoS Biol. 2010 Mar 23;8(3):e1000339. doi: 10.1371/journal.pbio.1000339 (PMC2843593; doi:10.1371/journal.pbio.1000339)
Supplement: Table S4 — Species and strains with genes for more than one pathway to lanthionine-containing peptides. Figure 6 in the main text shows species with genes for more than one pathway to lanthionine-containing peptides. In some cases, these genes are found in one strain; in other cases, the genes are found in different strains. For accession numbers, see Table S3. (0.01 MB PDF) [file pbio.1000339.s013.pdf]

Multiple lanthionine biosynthetic genes from different pathways found in a single strain

Streptomyces griseus subsp. griseus NBRC 13350;	LanB and LanL
Streptomyces clavuligerus ATCC 27064;	LanB and LanL
Geobacillus thermodenitrificans NG80-2;	LanB and LanM
Bacillus halodurans C-125;	LanM and LanB
Streptococcus pyogenes MGAS10750;	LanM and LanB

Examples of lanthionine biosynthetic genes for different pathways found in different strains in a species

Streptococcus pneumoniae CDC1087-00;	LanL
Streptococcus pneumoniae ATCC 700669;	LanMs
Streptococcus pneumoniae CGSP14;	LanB
Lactococcus lactis subsp. lactis C2102	LanM
Lactococcus lactis subsp. lactis 6F3	LanB

NOTE: in cases where the genome of the strain has not been fully sequenced, the current information is necessarily incomplete and multiple pathways could be present.
