# Supplementary material for: Discovery of Unique Lanthionine Synthetases Reveals New Mechanistic and Evolutionary Insights
Source: PLoS Biol. 2010 Mar 23;8(3):e1000339. doi: 10.1371/journal.pbio.1000339 (PMC2843593; doi:10.1371/journal.pbio.1000339)
Supplement: Text S1 — Molecular biology procedures, antimicrobial assays, and enzyme expression and purification protocols. Description of procedures used to construct the plasmids VenL/pET28, VenL-ΔC/pET28, VenL-ΔLC/pET28, VenL-ΔKC/pET28, and MBP-VenA/pET28. Also provided are the expression and purification protocols for these proteins and the methods used for site-directed mutagenesis of venA. Construction of the ΔvenL, ΔvenA, and ermEp*::venL derivatives of S. venezuelae are also described as well as antimicrobial assays with venezuelin and its analogs. (0.09 MB PDF) [file pbio.1000339.s014.pdf]

Supporting information

Discovery of Novel Lanthionine Synthetases Provides Mechanistic and Evolutionary Insights

Yuki Goto[1], Bo Li[2], Jan Claesen[3], Yanxiang Shi,[1] Mervyn J. Bibb[3], and Wilfred A van der Donk[1,2,4*]

[1]Department of Chemistry, [2]Department of Biochemistry, and [4]the Howard Hughes Medical Institute, University of Illinois at Urbana-Champaign, 600 S. Mathews Ave., Urbana, Illinois 61801, USA.
[3]Department of Molecular Microbiology, John Innes Centre, Norwich NR4 7UH, United Kingdom

[*]To whom correspondence should be addressed. E-mail: vddonk@illinois.edu.

Supporting materials and methods

Materials. All oligonucleotides were purchased from Operon Technologies, Integrated DNA Technologies, or Sigma-Aldrich. Restriction endonucleases, DNA polymerases, and T4 DNA ligase were purchased from Invitrogen or New England Biolabs. Media for bacterial culture and chemicals were purchased from Difco laboratories, CalBiochem, Aldrich, or Fisher Scientific unless noted otherwise and used without further purification.

Strains and plasmids. *S. venezuelae* ATCC10712 and *S. lividans* TK24 were maintained as previously described [1]. *E. coli* DH5α was used as host for cloning and plasmid propagation, and *E. coli* BL21 (DE3) was used as a host for protein expression. *E. coli* BW2113/pIJ790 and *E. coli* ET12567/pUZ8002 were used as before [2]. *Micrococcus luteus* ATCC4698 was employed as indicator strain for the antibiotic activity assays. Cloning vectors (pET15 and pET28) were obtained from Novagen. MBP/pET28 was kindly provided by Dr. Jack E. Dixon (UCSD) [4]. pIJ773 (1) and pIJ10702, also known as pMJCos1 [3], have been described. The over-expression cassette was generated by fusing the apramycin resistance and *oriT* cassette of pIJ773 to the constitutive *ermE** promoter and EF-Tu ribosome binding site by overlapping PCR. Cloning of this extended cassette into pGEM-T Easy (Promega) yielded pIJ10704 (J.C. and S. O'Rourke, unpublished data).

General methods. All polymerase chain reaction (PCR) amplifications were carried out with an automated thermocycler (PTC-100 or PTC-150, MJ Research). DNA sequencing was conducted using the appropriate primers by the Biotechnology Center of the University of Illinois at Urbana-Champaign. MALDI-ToF MS and ESI-Q/ToF MS were carried out on a Voyager-DE-STR (Applied Biosystems) and a Synapt MS system equipped with an Acquity UPLC (Waters), respectively.

Construction of VenL/pET28, VenL-ΔC/pET28, VenL-ΔLC/pET28, and VenL-ΔKC/pET28. PCR amplification of *venL* was performed by thirty cycles of denaturing (94 °C for 30 s), annealing (58 °C for 45 s), and extending (72 °C for 120 s) using the appropriate primers (see Table S2) and using the 4H08 cosmid described below as DNA template. VenL_*Nde*I_FP and VenL_*Hind*III_RP were used for cloning *venL*, VenL_*Nde*I_FP and VenL-ΔC_*Hind*III_RP were used for generating the sequence encoding VenL-ΔC,

VenL-ΔLC_*Nde*I_FP and VenL-ΔC_*Hind*III_RP were used for generating the sequence encoding VenL-ΔLC, and VenL_*Nde*I_FP and VenL-ΔKC_*Hind*III_RP were used for generating the sequence encoding VenL-ΔKC. The PCR mixtures included 1× FailSafe PreMix G (PICENTRE Biotechnologies), DMSO (4%), Platinum *Pfx* DNA polymerase (0.025 U/μL), *Taq* DNA polymerase (0.05 U/μL), and primers (1 μM each). Amplifications were confirmed by 2% agarose gel electrophoresis, and the products were purified using QIAquick PCR Purification Kits (QIAGEN). The resulting DNA fragments and the pET28 vector were digested in 1× NEBuffer 2 (New England Biolabs) with *Nde*I and *Hind*III at 37 °C for 15 h. The reaction mixtures were purified by agarose gel electrophoresis followed by QIAquick Gel Extraction Kit (QIAGEN). The resulting DNA inserts were ligated individually with the digested pET28 vector at 24 °C for 3 h using T4 DNA ligase. The ligation reaction mixtures were diluted 10 times with water prior to transformation. *E. coli* DH5α cells were transformed with the ligation product via heat shock and were plated on LB-kanamycin agar plates and grown at 37 °C for 15 h. Three colonies were picked up and incubated in 5 mL of LB-kanamycin medium at 37 °C for 15 h, followed by isolation of the plasmids using a QIAprep Spin Miniprep Kit (QIAGEN). The sequences of the resulting plasmids were confirmed by DNA sequencing.

Construction of MBP-VenA/pET28. Cloning of *venA* into pET15 was carried out using a similar procedure as that described in the previous paragraph. PCR amplification of *venA* was performed by thirty cycles of denaturing (94 °C for 20 s), annealing (58 °C for 30 s), and extending (72 °C for 45 s) using the VenA_*Nde*I_FP and VenA_*Xho*I_RP primers (see Table S2) and with the 4H08 cosmid described below as DNA template. The resulting PCR product and the pET15 vector were digested with *Nde*I and *Xho*I, ligated and used to transform *E. coli* DH5α cells. The sequence of the resulting plasmid isolated from DH5α was confirmed by DNA sequencing.

PCR amplification of *venA* fused with a hexahistidine tag sequence was performed using pET15_*Bam*HI_FP and VenA_*Not*I_RP primers (see Table S2 for the sequences). The resulting PCR product and the MBP/pET28 vector were digested with *Bam*HI and *Not*I, ligated and used to transform *E. coli* DH5α cells. The sequence of the resulting plasmid isolated from DH5α was confirmed by DNA sequencing.

Site-directed mutagenesis of VenA. Site-directed mutagenesis of *venA* was performed by multistep PCR. First, *venA-A29K* was constructed via Quikchange (Stratagene). The primers used were A29K_FP and A29K_RP (see Table S2 for the sequences). The PCR mixture included 1× FailSafe PreMix G (PICENTRE Biotechnologies), *Pfu Turbo* DNA polymerase (0.5 U/μL, Stratagene), *venA*/pET15 (2 ng/μL), and primers (1.4 μM each). PCR reaction was conducted by thirty cycles of denaturing (95 °C for 1 min), annealing (52 °C for 1 min), and extending (68 °C for 8 min). A sample of 5 μL PCR product was checked on a 1% agarose gel, and 1 μL of *Dpn*I was added to the rest of the PCR product and incubated at 37 °C for 4 h to digest the methylated template. A sample of 10 μL *Dpn*I digested product was used to transform DH5α cells. The desired mutations were confirmed by DNA sequencing.

Next, the amplification of *venA-A29K* was carried out by thirty cycles of denaturing (94 °C for 20 s), annealing (58 °C for 30 s), and extending (72 °C for 45 s) using pET15_*Bam*HI_FP primer and mutant reverse primers (C32A_RP, C34A_RP, C45A_RP, or C50A_RP) (see Table S2 for the sequences) to yield 5' fragments of the mutated *venA* genes (FP reaction). The PCR mixture included 1× PCR reaction buffer, dNTPs (10 mM each), $MgCl_2$ (1.5 mM), *Taq* DNA polymerase (0.05 U/μL), and primers (1 μM each). In parallel, PCR reactions using mutant forward primers (C32A_FP, C34A_FP, C45A_FP, or C50A_FP)

and the appropriate reverse primers (VenA_*Not*I_RP primer for C32A, C34A, and C45A mutations, VenA_C50A_*Not*I_RP for C50A mutation) were also conducted to produce 3' fragments of the mutated *venA* genes (RP reaction). The overlapping products from the FP reaction and RP reaction were combined in equal amounts and extended by five cycles of denaturing, annealing, and extending in a solution containing 1× FailSafe PreMix G (PICENTRE Biotechnologies), DMSO (4%), and Platinum *Pfx* DNA polymerase (0.025 U/μL). Following the extension, the appropriate primers were added (pET15_*Bam*HI_FP and VenA_*Not*I_RP for C32A, C34A, and C45A mutations, pET15_*Bam*HI_FP and VenA_C50A_*Not*I_RP for C50A mutation; final concentration, 2 μM), and the reaction mixture was incubated for 25 additional cycles of denaturing, annealing, and extending. The final PCR products were purified by agarose gel electrophoresis and cloned into MBP/pET28. The desired mutations were confirmed by DNA sequencing.

Overexpression and purification of His$_6$-VenL, His$_6$-VenL-ΔC, His$_6$-VenL-ΔLC, and His$_6$-VenL-ΔKC. BL21(DE3) cells transformed with a pET28 vector carrying each gene were grown in 2 L of LB medium containing 50 mg/L kanamycin at 37 °C until the OD_{600nm} reached about 0.6. The incubation temperature was then changed to 18 °C and the culture was induced with 0.2 mM IPTG. The induced cells were shaken continually at 18 °C for an additional 18 h. The cells were harvested by centrifugation (11,900 × *g* for 10 min, Beckman JLA-10.500 rotor). The cell pellet was resuspended in 30 mL of start buffer (50 mM HEPES·Na, pH 7.4, 300 mM NaCl, 10% glycerol, containing a protease inhibitor cocktail from Roche Applied Science) and stored at –80 °C.

All protein purification steps were performed at 4 °C. Cell paste in start buffer was sonicated on ice for 20 min. After centrifugation (23,700 × *g* for 30 min, Beckman JA-20 rotor), the supernatant was filtered through a 0.45 μm syringe filter. The sample was then loaded onto a 5 mL HisTrap HP column (GE Healthcare Life Sciences). The column was washed with 20 mL each of start buffer containing 25 mM and 50 mM imidazole and then 10 mL each of start buffer containing 100 mM, 200 mM, and 500 mM imidazole. The eluent was collected in several fractions, which were analyzed by Tris-SDS PAGE. The fractions containing the desired protein (200 mM–500 mM imidazole) were combined and concentrated using an Amicon Ultra-15 Centrifugal Filter Unit (30 kDa Mw cut off for His$_6$-VenL, 10 kDa Mw cut off for others, Millipore) to less than 2 mL. Buffer exchange of the concentrated protein with start buffer was carried out twice using a PD-10 desalting column (GE Healthcare Life Sciences). The resulting protein sample was stored at –80 °C. Protein concentrations were determined using a BCA Protein Assay Kit (Pierce Biotechnology), demonstrating protein yields of 5.5 mg, 11 mg, 2.4 mg, and 30 mg for His$_6$-VenL, His$_6$-VenL-ΔC, His$_6$-VenL-ΔLC, and His$_6$-VenL-ΔKC, respectively, from 2 L of cell culture.

Overexpression and purification of His$_6$-VenA and its mutant variants. BL21(DE3) cells transformed with each MBP-substrate/pET28 plasmid were grown in 2 L of LB medium containing 50 mg/L kanamycin at 37 °C until the OD_{600nm} reached 0.5. Then, IPTG was added to the culture (final concentration 0.3 mM), and the cells were continually shaken at 37 °C for an additional 2.5–3 h. Cells were harvested by centrifugation (11,900 × *g* for 10 min), and the cell pellet resuspended in 30 mL of start buffer and stored at –80 °C.

All protein purification steps were performed at 4 °C. Cell paste was suspended in start buffer and the resulting suspension was sonicated on ice for 20 min. After centrifugation (23,700 × *g* for 30 min), the supernatant was filtered through a 0.45 μm syringe filter. The sample was then loaded onto a 5 mL HisTrap HP column. The column was washed with 40 mL of start buffer containing 100 mM imidazole and then 20 mL of start buffer containing 500 mM

imidazole. The eluate was collected into several fractions, which were analyzed by Tris-SDS PAGE. The fractions containing the MBP-fusion protein (500 mM imidazole eluate) were combined and concentrated using an Amicon Ultra-15 Centrifugal Filter Unit (30 kDa Mw cut off) to less than 2 mL. Buffer exchange of the concentrated protein with start buffer was carried out twice using a PD-10 desalting column and the protein sample was stored at –80 °C.

The resulting MBP-fusion protein (875 μL) was incubated with tobacco etch virus (TEV) protease in 1.6 mL of TEV buffer (50 mM HEPES, pH 7.0, 0.5 mM EDTA, 1 mM DTT) at 30 °C overnight. The precipitated peptide was collected by centrifugation (21,100 × *g* for 15 min), and washed with 30 μL of water and centrifuged again. The peptide was dissolved in 5–20 mL of water/MeCN and the solution lyophilized. The resulting powder was dissolved in DMSO to generate a stock solution (600 μM).

Construction of Δ*venL*, Δ*venA* and *venL* over-expression mutants of *S. venezuelae*. A SuperCosI derivative (4H08) containing the *ven* gene cluster (M.J.B, unpublished data) was used as template for PCR-targeting [2]. To generate the Δ*venL* and Δ*venA* deletion mutants, each gene present in cosmid 4H08 was replaced by an apramycin resistance cassette from pIJ773 that was amplified with appropriate flanking primers (RD_VenL_*SpeI*_FP and RD_VenL_*XbaI*_RP for Δ*venL* and RD_VenA_*SpeI*_FP and RD_VenA_*XbaI*_RP for Δ*venA*; see Table S2 for the sequences). Amplification of the over-expression cassette from pIJ10704 was achieved using primers RD_VenL_*ermE*p_FP and RD_VenL_*ermE*p RP (Table S2). Targeting of cosmid 4H08 with this cassette resulted in the insertion of the constitutive *ermE** promoter and EF-TU ribosome binding site directly upstream of the start codon of *venL*. All cosmid constructs were conjugated into *S. venezuelae* and the required double cross-over mutants (subsequently confirmed by PCR) obtained by screening for retention of apramycin resistance but loss of the vector-encoded kanamycin resistance. The wild type 4H08, 4H08 Δ*venL* and 4H08 Δ*venA* cosmids were targeted with the *Ssp*I fragment of pIJ10702, and the resulting constructs conjugated into *S. lividans* TK24. Apramycin-resistant exconjugants were verified by PCR. Liquid-grown cultures of *S. lividans* were analyzed by MALDI-ToF, using both culture supernatants (undiluted and concentrated) and mycelial extracts.

Antibiotic activity assays. *S. venezuelae* and the Δ*venL*, Δ*venA* and *venL* over-expression derivatives, as well as *S. lividans* TK24 derivatives containing the integrated vector (pIJ10702), the wild type, Δ*venL* and Δ*venA* gene clusters were inoculated onto 10 different agar media and incubated at 30 °C for five days. In addition, induction of the cluster with N-acetylglucosamine and S-adenosylmethionine, which have both been described as activating cryptic gene clusters in streptomycetes, was attempted. Antibiotic activity was assayed by overlaying the strains with *M. luteus* ATCC4698 in Soft Nutrient Agar (SNA) medium [1] and analyzing for zones of growth inhibition. The overlay was prepared by diluting (1:8) a rapidly growing culture of *M. luteus* (OD_{600} of ~0.8) into 12 mL aliquots of SNA, which were subsequently used to overlay each agar plate containing the *Streptomyces* strains. The plates were incubated at 30°C and scored for zones of inhibition after 48 hours.

Antibiotic activity assays of venezuelin analogs prepared *in vitro* were also performed. VenA mutants (9 nmol) containing protease-recognition sites between leader peptide and propeptide region were incubated with VenL followed by incubation with protease as previously described. The resulting peptide was desalted by using a solid phase extraction column (Discovery DSC-18 SPE, Aldrich) and resuspended in DMSO. The solution was spotted on agar plates containing *B. subtilis* LH45, *L. lactis* HP, or *M. luteus* ATCC4698. The plates were incubated at the proper temperature (30°C for *L. lactis* HP, 37°C

for *B. subtilis* LH45 and *M. luteus* ATCC4698) and the plates were checked for zones of growth inhibition after 15 hours (Figure S5).

Amino acid sequence of VenL.
MTSRVTEVELEGLLREALHATGTGARWAVEADEMWCRVTPGAGTRRDQGWKLHLS
ATAASAPAVLEKALGVLLREESPFKFARSLDQVSALNSRATPLGSSGKFITVYPRSDAG
AARLAHELHGATAGLAGPRILSDQPYAVNSLVHYRYGSFVGRRRLSDDGLLVWFIEDP
DGNPVEDQRTGRYAPPPWAVCPFPATVPVVPRAAEGGQGADGSREARGAGAPVLLG
GRFAVREAIRHTNKGGVYRGSDVRTGALVVIKEARPHVEADASGHDVRDWLRTEAR
TLERLRGTGLAPEPLALFEHGGHLFLAQQEVPGVALRTWVAEHFRDAGAERYRVDA
RAQVGRLVELVAAAHAHGCVLRDFTPGNVMVRPDGELRLIDLELAVLEGDAARPTR
VGTPGFSAPERLTGAPVSPTGDYYSLGATACFVLAGKVPNLLPEEPATRAPEQRLAAW
LSACAGPLRLPDGLAEMVLGLMKDAPAERWDPARAREALRRADRDDPVTGKPEDS
KGSKGSQAGNVGDAVTVNVGGTVARPLAHPTPGTASAAGADDIAVGVDEAVAGIVD
HLLDAMTPADERRLWPVSTMAGETDSCTVQQGAAGVLGVLTRYWELTGDDRLPELI
STAGHWIAHRTDLSSTRPGLHFGGRGTAWALYDAGRAVDDDRLMAHATALALAPQQ
STPHHDITHGTAGSGVAAVHFWHRTGDPRFAELVVDAADRLTAAGRRDRSGVGWPV
PAEAASPEGGTGYLGFAHGTAGIGCFLLAAAAVSGRPEHLDLAVEAGEHLRDQAVLT
GEAAHWPARSADPPTAAYWCHGAAGIGTFLVRLWQATGDDRFGELARRATHAVTER
ASRAALAQCHGLAGNGDFLLDMADATGDPVHRTAAEDLARLLLGERAHRRAQVVF
PNEYGDVSTSWSDGSAGILAFLLRIRHADPRHWMVQRPVRAAAGLSPAH.

Supporting Information References

1. Keiser T *et al.* (2003) Practical *Streptomyces* Genetics. Norwich, UK: The John Innes Foundation.
2. Gust B *et al.* (2004) Lambda *red*-mediated genetic manipulation of antibiotic-producing *Streptomyces*. Adv Appl Microbiol 54: 107-128.
3. Boakes S *et al.* (2009) Organization of the genes encoding the biosynthesis of actagardine and engineering of a variant generation system. Mol Microbiol 72: 1126-1136.
4. Lee SW, Mitchell DA, Markley AL, Hensler ME, Gonzalez D, et al. (2008) Discovery of a widely distributed toxin biosynthetic gene cluster. Proc Natl Acad Sci USA 105: 5879-5884
5. Widdick DA, Dodd HM, Barraille P, White J, Stein TH, et al. (2003) Cloning and engineering of the cinnamycin biosynthetic gene cluster from Streptomyces cinnamoneus cinnamoneus DSM 40005. Proc Natl Acad Sci USA 100: 4316-4321.
